# Overexpression of olfactory receptor 78 ameliorates brain injury in cerebral ischaemia–reperfusion rats by activating Prkaca‐mediated cAMP/PKA‐MAPK pathway

**DOI:** 10.1111/jcmm.18366

**Published:** 2024-06-10

**Authors:** Tao Kang, Lijuan Zhu, Yanli Xue, Qian Yang, Qi Lei, Qianqian Wang

**Affiliations:** ^1^ Department of Neurology Shaanxi Provincial People's Hospital Xi'an China; ^2^ Department of Anesthesia Shaanxi Provincial People's Hospital Xi'an China; ^3^ Department of Traditional Chinese Medicine The First Affiliated Hospital of Xi'an Jiaotong University Xi'an China

**Keywords:** Ca^2+^ deposition, ischemic stroke, Olfr78, Prkaca, the cAMP/PKA‐MAPK signalling pathway

## Abstract

Ischemic stroke is one of the main causes of disability and death. However, recanalization of occluded cerebral arteries is effective only within a very narrow time window. Therefore, it is particularly important to find neuroprotective biological targets for cerebral artery recanalization. Here, gene expression profiles of datasets GSE160500 and GSE97537 were downloaded from the GEO database, which were related to ischemic stroke in rats. Olfactory receptor 78 (Olfr78) was screened, and which highly associated with Calcium signalling pathway and MAPK pathway. Interacting protein of Olfr78, Prkaca, was predicted by STRING, and their interaction was validated by Co‐IP analysis. Then, a rat model of middle cerebral artery occlusion/reperfusion (MCAO/R) and a neuronal cell model stimulated by oxygen–glucose deprivation/reoxygenation (OGD/R) were constructed, and the results showed that expression of Olfr78 and Prkaca was downregulated in MCAO rats and OGD/R‐stimulated neurons. Overexpression of Olfr78 or Prkaca inhibited the secretion of inflammatory factors, Ca^2+^ overload, and OGD/R‐induced neuronal apoptosis. Moreover, Overexpression of Prkaca increased protein levels of cAMP, PKA and phosphorylated p38 in OGD/R‐stimulated neurons, while SB203580, a p38 inhibitor, treatment inhibited activation of the cAMP/PKA‐MAPK pathway and counteracted the effect of Olfr78 overexpression on improvement of neuronal functions. Meanwhile, overexpression of Olfr78 or Prkaca markedly inhibited neuronal apoptosis and improved brain injury in MCAO/R rats. In conclusion, overexpression of Olfr78 inhibited Ca^2+^ overload and reduced neuronal apoptosis in MCAO/R rats by promoting Prkaca‐mediated activation of the cAMP/PKA‐MAPK pathway, thereby improving brain injury in cerebral ischaemia–reperfusion.

## 1 INTRODUCTION

Stroke is the second leading cause of long‐term severe disability and death worldwide. Approximately 15 million people experience stroke every year, with 87% of ischemic stroke.^1^, ^2^ Recently, mechanical thrombectomy (MT) has significantly improved the success rate of recanalization and improved neurological sequelae after acute thrombosis occlusion of the internal carotid artery or middle cerebral artery (MCA).^3^, ^4^ Although reperfusion after cerebral ischemia cloud timely restore the blood circulation in ischemic tissues, it may lead to neuronal cell injury and death, known as cerebral ischaemia–reperfusion injury (CIRI).^5^ Neuroprotection aims to enable neurons to survive in hypoxia for a long time, but the long‐term survival of neurons clearly requires the reestablishment of blood and oxygen supply.^5^, ^6^ Therefore, it's urgent need to identify novel and effective therapeutic targets and the underlying molecular mechanisms of cerebral ischemia in stroke patients.

Odour receptors (OR) constitute a superfamily of G protein coupled receptors (GPCRs). Their expression in tissues other than olfactory epithelial cells has been extensively studied and increasingly reported.^7^, ^8^ Olfactory receptor 78 (Olfr78), as a member of GPCRs, has been identified the mainly receptor for Short‐chain fatty acids (SCFAs),^9^ which is widely expressed, such as in the brain tissues,^10^ kidneys,^11^ arterioles,^12^ carotid bodies,^13^ macrophages,^14^ colon,^15^ and prostate.^16^ Early reports showed that SCFAs inhibit vascular calcification by regulating the osteogenic reprogramming of vascular smooth muscle cells, thereby alleviating chronic kidney injury and atherosclerosis.^17^, ^18^, ^19^ Similarly, Olfr78 is thought to sense various metabolic byproducts of anaerobic cell respiration or bacterial fermentation, which is associated with hypoxia related reactions in the kidneys and carotid body, as well as bacterial metabolite sensing and hormone secretion in the colon.^13^, ^20^, ^21^ In addition, SCFAs improve cardiovascular disease progression by participating in blood pressure regulation.^22^ Olfr78 was also found to be expressed in the soma and axons of vasopressin/oxytocin neurons in the paraventricular/supraoptic nucleus of the hypothalamus, and moderately expressed in microglia near the parenchymal vasculature.^10^ The above studies suggest that Olfr78 may be closely related to cardiovascular disease. However, the role of Olfr78 in neuronal damage caused by cerebral ischemia remains unclear.

Prkaca gene codes for the Cα catalytic subunit of protein kinase A (PKA‐Cα). PKA is a cyclic AMP (cAMP) dependent protein kinase, consisting of a tetramer consisting of two regulatory (R) subunits and two catalytic (C) subunits that containing active kinase sites.^23^ There are three isomers of PKA catalytic subunit, of which Cα is considered the major subtype and is expressed in most tissues.^23^, ^24^ Deletion of the Prkaca gene leads to growth retardation in a few surviving animals, and Prkaca deficiency is also associated with spinal canal defects.^25^ The increase of the second messenger cAMP in the cytoplasm leads to binding to the regulatory subunit, and subsequently the catalytic subunit of PKA was dissociated and activated.^26^ Free catalytic subunits are subsequently phosphorylated by proteins and are involved in many cellular processes, such as metabolism, synaptic transmission, differentiation, growth, and development.^26^, ^27^, ^28^ Existing reports have shown that compared with the control group, the neurological scores of CIRI rats were increased, neuronal apoptosis in the brain tissues was significantly increased, and the expression of cAMP and PKA in brain tissues were decreased, while lidocaine improved CIRI in rats through promoting the expression of cAMP and PKA.^29^ Meanwhiles, teriparatide reduced CIRI in rats by inducing angiogenesis in ischemic cerebral infarction zones^30^; Senkyunolide H inhibited oxygen‐glucose deprivation/reoxygenation (OGD/R)‐induced injury of PC12 cells via activation of the cAMP‐PI3K/AKT signalling pathway^31^; the activation of cAMP‐dependent protein kinase (PKA) has a neuroprotective against transient cerebral ischemia.^32^ Therefore, we determined that Prkaca plays a key role in CIRI.

With the progress of high‐throughput sequencing technology, RNA‐Seq has become a powerful tool for identifying biomarkers in diseases. Therefore, in this study, we obtained the target gene Olfr78 by screening differentially expressed genes (DEGs) form the GSE160500 and GSE97537 datasets. Then, interacting protein of Olfr78, Prkaca, was predicted by the STRING database (https://cn.string‐db.org/), and their interaction was validated by Co‐immunoprecipitation (Co‐IP) analysis. In addition, OGD/R‐induced neuronal cell model in vitro and rat middle cerebral artery occlusion/reperfusion (MCAO/R) model in vivo were constructed, and the role of Olfr78 and Prkaca in CIRI and its potential regulatory mechanism were explored through loss‐ and gain‐of‐function experiments. The above findings can provide molecular relevant insights into the pathogenesis of CIRI and provide strategies for screening therapeutic targets related to CIRI.

## 2 MATERIALS AND METHODS

### 2.1 Bioinformatics analyses for datasets from GEO

The datasets GSE160500 and GSE97537 from the GEO database (https://www.ncbi.nlm.nih.gov/geo/) were screened, and RNA data was downloaded for further analysis. Six samples were selected from GSE610500, including 3 MCAO/R model rats and 3 Sham rats, and 12 samples were selected from GSE97537, including 7 MCAO/R model rats and 5 Sham rats. GSE97537 dataset is from Sprague Dawley rats, which belongs to GPL1355 platform; The sequencing information of GSE160500 dataset is from adult male Wistar rates, which belongs to GPL22396 platform.

Briefly, fastp software (v0.20.0) was used to trim adaptor and remove low quality reads to get high‐quality clean reads. STAR software (v2.7.9a) was used to align the high‐quality clean reads to the human reference genome (hg38). The featureCounts software (v2.0) was used to get the raw gene level mRNA read counts as the mRNA expression profile. DESeq2 software (v1.30.1) was used to normalize and calculate the fold change and *p*‐value between two groups. A volcanic map was drawn using R package ggplot2. A heat map of differentially expressed genes was drawn using R package pheatmap. Use the R package ClusterProfiler to perform Gene Ontology (GO) and Kyoto Encyclopedia of Genes and Genomes (KEGG) pathway enrichment analysis on differentially expressed genes (DEGs). In addition, the online bioinformatic tool Jvenn was used to intersect DEGs among the GSE160500 dataset and GSE97537 dataset. GO and KEGG pathway enrichment analysis were performed with the clusterProfiler R package (v3.18.1) based on the DEGs.

### 2.2 Clinical case collection

In this study, a total of 124 patients (57.3 ± 11.5 years) who were diagnosed with acute ischemic stroke in Shaanxi Provincial People's Hospital (Xi'an, China) from June 2020 to February 2023 were included. The inclusion criteria were the first confirmation of ischemic stroke by MRI within 24 h of onset, and the severity of ischemic stroke at admission was assessed according to the National Institutes of Health Stroke Scale [NIHSS] scores (modified Rankin Scale [mRS] scores, 0–1; range, 0 [no symptoms] to 6 [death], with a higher score indicating worse symptoms).^33^ The exclusion criteria were as follows: history of previous stroke, history of other disorders of the nervous system, allergy to radiographic contrast agents, disability prior to stroke occurrence (modified Rankin Scale score >3), pregnant or breastfeeding women, diagnosis 24 h after onset, receipt of thrombolytic therapy, and incomplete information required for analysis. Patients were categorized according to the expression levels of OR51E2/Olfr78 and Prkaca in the venous blood of patients on the 24 h of admission. The characteristics of patients included gender, age, hypertension or not, diabetes mellitus or not, smoking or not, drinking or not, and serum levels of total cholesterol (TC), triglyceride (TG), low‐density lipoprotein cholesterol (LDL‐C), and high‐density lipoprotein cholesterol (HDL‐C). All participants obtained informed consent. This study was approved by the Research Ethics Review Committee of Shaanxi Provincial People's Hospital.

### 2.3 Cell lines and culture

As mentioned earlier, primary neuronal cells were derived from newborn SD rats.^34^ Briefly, rat cortical tissues were isolated from embryonal rat brain tissues (aged 16–18 days) and maintained in ice‐cold PBS, followed by digestion with 0.25% trypsin (Gibco, Waltham, MA, USA) at 37°C for 10 min. Next, cells were suspended in DMEM/F12 medium (Gibco, Waltham, MA, USA) containing 10% FBS, and then centrifugated at 200 *g* for 5 min at 4°C. Subsequently, cells were resuspended in DMEM/F12 medium at s seeding density of 5 × 10^4^ cells/well on a poly‐D lysine coated plate, at 37°C and 5% CO_2_ for 4 h, and then replaced with serum‐free neuroblastoma A medium (Gibco) containing 2% B27.

### 2.4 Construction of oxygen–glucose deprivation and reperfusion model

The OGD/R model was constructed in vitro to simulate MCAO/R. Isolated primary neuronal cells were cultured in the presence of glucose and serum‐free medium at 37°C in an anaerobic chamber containing 5% CO_2_ and 95% N_2_. After 2 h, the cells were transferred to normal culture medium and continued to be cultured under normal conditions for 24 h.

### 2.5 Cell transfection

Olfr78 overexpression vector (pcDNA‐Olfr78) and shRNA (sh‐Olfr78), Prkaca overexpression vector (pcDNA‐Prkaca) and their control were designed and synthetized from GenePharma Co., Ltd. (Shanghai, China). Next, cells were plated into six‐well plates for 24 h, transfected when the cells fused to about 60%, and harvested 48 h later. All transfection was performed with Lipofectamine®3000 (Thermo, Waltham, MA, USA) according to manufacturer's instructions.

### 2.6 Cell viability assay

The neuronal viability was measured by using a Cell Counting Kit‐8 kit (CCK‐8) assay (Sigma‐Aldrich, St. Louis, MO, USA). Briefly, neuronal cells were seeded into 96‐well plates and incubated for 48 h, and then 10 μL of CCK‐8 solution was added to each well and incubated at 37°C for 2 h in a humidified atmosphere with 5% CO_2_. The absorbance of each well at 450 nm was measured through a microplate reader (Molecular Devices, Shanghai).

### 2.7 Cell apoptosis assay

Cell apoptosis was detected by using a FACS Aria Sorter (Becton Dickinson, San Jose, CA, USA). The cells were resuspended in 500 μL of buffer solution (PBS with calcium) and mixed with 5 μL of Annexin V‐FITC and 10 μL of propidium iodide (PI) for 15 min in the dark. Following incubation, 400 μL of binding buffer was added to each sample, and cell apoptosis was analysed with flow cytometry (Becton, Dickinson and Company, Franklin Lakes, New Jersey, USA). The data were analysed using FlowJo 7.6 software (FlowJo, LLC, Ashland, OR, USA). Annexin V‐positive cells were apoptotic cells, and the right upper quadrant and the right lower quadrant presented the apoptotic cells.

### 2.8 Construction of middle cerebral artery occlusion reperfusion model

Seventy‐two adult male SD rats, aged 6–8 weeks and weighing 250–280 g, were obtained from the experimental animal centre of Xi'an JiaoTong University. All animals were housed in an environment with a temperature of 22 ± 1°C, a relative humidity of 50 ± 1%, and a light/dark cycle of 12/12 h for 10 days, and were allowed to drink and eat freely. The experiment was conducted according to the Guide for Care and Use of Laboratory Animals of the National Institutes of Health and approved by the Ethics Committee of the Shaanxi Provincial People's Hospital (Approval number: SPPH‐2022081).

Forty adult male SD rats were randomly divided into five groups: sham group, MCAO/R group, MCAO/R + LV‐NC group, MCAO/R + LV‐Olfr78 group, and MCAO/R + LV‐Prkaca group, with 8 rats in each group. All animals were anaesthetised by intraperitoneal injection of 10% chloral hydrate (30 mg/kg). According to the intraluminal vascular occlusion method depicted previously, the MCAO/R model was established, with some adjustment.^35^ Briefly, a 4–0 surgical nylon segment with a length of 18.0–19.0 mm (based on animal weight measurement) was slowly advanced from the left common carotid artery to the lumen of the internal carotid artery until reaching the proximal end of the anterior cerebral artery. After 90 min of MCAO, ~5 mm of nylon suture was removed to achieve reperfusion. During the operation, physiological factors, including body temperature, were observed. Generally, all animals were treated with 90 min of transient MCAO with the aim of producing reproducible and consistent ischemic injury in the cortex and unilateral striatum. A burr hole was drilled into the pericranium. A 10‐mL syringe was inserted into the basal ganglia 3 mm under the meninges. Lentivirus carrying Olfr78 overexpression vector (LV‐Olfr78), Prkaca overexpression vector (LV‐Prkaca) or control (LV‐NC) (5 × 10^9^/100 μL) were injected into the hemisphere 90 min before the MCAO. All animals were euthanized 48 h after MCAO/R, and blood and brain tissues were collected for further experiments.

Thirty‐two adult male SD rats were randomly divided into four groups, the sham group, MCAO/R model group, MCAO/R + Propionate group, and the MCAO/R+ Propionate+ DS89002333 group, with 8 rats in each group. All animals were anaesthetised by intraperitoneal injection of 10% chloral hydrate (30 mg/kg), and the MCAO/R model was established according to the above method of the intraluminal vascular occlusion. Rats were orally administered sodium propionate (300 mg/kg) as the MCAO/R + Propionate group, and Rats were orally administered sodium propionate (300 mg/kg) and DS89002333 (80 mg/kg) as the MCAO/R+ Propionate+ DS89002333 group. Rats of the MCAO/R + Propionate group and the MCAO/R + Propionate+DS89002333 group were administrated ig once a day after MCAO/R for 7 days. The sham group and MCAO/R model group were treated with normal saline. After 7 days of modelling, the neurological score of each rat was determined. Then, the rats were euthanized. The brain tissues were separated from rats and frozen in liquid nitrogen to await subsequent analysis.

### 2.9 2,3,5‐Triphenyltetrazolium chloride staining and quantification of infarct volume

After 48 h of ischaemia–reperfusion, rats were sacrificed and brain tissues were rapidly collected, snap frozen at −20°C for 20 min, and then cut into 2‐mm thick tissue sections. And then, sections were incubated with 2% 2,3,5‐Triphenyltetrazolium Chloride (TTC) solution (Jiancheng Biotechnology) in the dark for 30 min at 37°C, followed by soak in 4% formalin for 4 h. Images of sections were taken and recorded after staining. Image J software was used to measure the infarct size and the entire area of each coronal section. The infarct volume was calculated as follow: infarct volume (%) = (V1 + V2 + … + V5)/(M1 + M2 + … + M5) × 100%, where V*n* represents the infarct volume of each section, and M*n* represents the total volume of each section.

### 2.10 TUNEL staining

Brain tissues were fixed with 4% paraformaldehyde, embedded in paraffin, and made into 5‐mm tissue sections. Then, TUNEL fluorescence assay was performed by using the Dead End Fluorometric TUNEL System kit (Promega Corp., Madison, WI, USA) according to the manufacturer's protocol. Subsequently, sections were stored in the dark before nuclear staining with the Hoechst fluorescence staining (Thermo Fisher Scientific, Waltham, MA, USA). Finally, TUNEL positive cells in the hippocampus were quantified by using NIS element image processing and analysis software (Nikon, Tokyo, Japan).

### 2.11 Ca^2+^ determination

Cells (1 × 10^6^) were harvested by centrifugation at 1200 × *g* for 5 min at room temperature, and Ca^2+^ was reacted with Infinity Calcium Arsenazo Liquid Stable Reagent to form a bluish‐purple coloured complex according to the manufacturer's instructions. The amount of colour formed was measured by increasing the absorbance of the reaction mixture at 600 and 660 nm by using FS5 spectrofluorometer.

### 2.12 Quantitative reverse transcription PCR (RT‐qPCR)

Total RNA was extracted from cerebral tissues and cells by using TRIzol reagent (Invitrogen, Carlsbad, CA, USA). Then, RNA was used as a template for reverse transcription to synthesize cDNA by using the RevertAid First Strand cDNA Synthesis Kit (#K1621, Thermo Scientific, USA). PCR amplification was performed using fluorescent dye SYBR™ Green PCR Master Mix (#43,091,055, Thermo Fisher, USA). Gene expression was quantified by calculating the 2^−ΔΔCt^ by normalizing the amount of internal control (β‐actin).

### 2.13 Western blotting

Brain tissues and cells were homogenized in a RIPA Lysis Buffer (Beyotime, China), and the concentration of the proteins was analysed by BCA kit (Solarbio, Beijing, China). Next, protein samples were separated in 12% SDS‐PAGE and transferred onto PVDF membranes. The membranes were prevented by the incubation with 5% non‐fat milk for 1 h at room temperature, and then incubated with primary antibodies, including Primary antibodies included Anti‐Olfr78 (1:1000, ab129883, Abcam), Anti‐Prkaca (1:1000, ab76238, Abcam), Anti‐cAMP (1:1000, ab134901, Abcam), Anti‐PKA (1:1000, ab313494, Abcam), Anti‐p38 (1:1000, ab182453, Abcam), Anti‐p‐p38 (1:1000, ab178867, Abcam), Bcl‐2 (1:1000, ab241548, Abcam), Caspase‐3 (1:1000, ab32351, Abcam), Ceaved‐caspases‐3 (1:1000, ab32042, Abcam), and β‐tubulin (1:1000, ab179513, Abcam) overnight at 4°C. On the following day, membranes were incubated with horseradish peroxidase (HRP)‐conjugated goat anti‐rabbit or goat anti‐mouse secondary antibodies for 2 h at room temperature. Finally, the membranes were emerged in the ECL reagent to visualize the immunoreactive bands and Fuji Image‐Gauge software (Fujifilm North America Corporation, Valhalla, NY, USA) was used to measure band intensity.

### 2.14 Co‐immunoprecipitation

Neuronal cells were rinsed with PBS and lysed in lysis buffer. Cell lysates were cleared by centrifugation for 5 min at 500 *g*, and the supernatants were incubated overnight with Anti‐Olfr78 antibody or Anti‐Prkaca antibody conjugated glutathione‐agarose beads. After overnight incubation, proteins attached to the beads were dissociated from the beads and loaded on SDS‐PAGE, transferred on PVDF membranes, and processed for detection of target proteins. The intensity of the bands was quantified by using ImageJ software (Fujifilm North America Corporation, Valhalla, NY, USA).

### 2.15 Enzyme‐linked immunosorbent assay

Serum and cell culture supernatant were assayed for Interleukin‐1β (IL‐1β), tumour necrosis factor alpha (TNF‐α), and Interleukin‐6 (IL‐6) with enzyme‐linked immunosorbent assay (ELISA0 kits Thermo Fisher Scientific, Waltham, Ma, USA), according to the manufacturer's instructions.

### 2.16 Statistical analysis

Quantitative data are presented as means ± SEM, and comparisons between two groups were performed by Student's *t*‐test. Statistical comparisons between multiple groups were evaluated using one‐way or two‐way analysis of variance (ANOVA) followed by the least significant difference (LSD) test. *p*‐value < 0.05 was considered statistically significant.

## 3 RESULTS

### 3.1 Olfr78 was screened as a potential regulator in progression of MCAO/R

**FIGURE 1:**
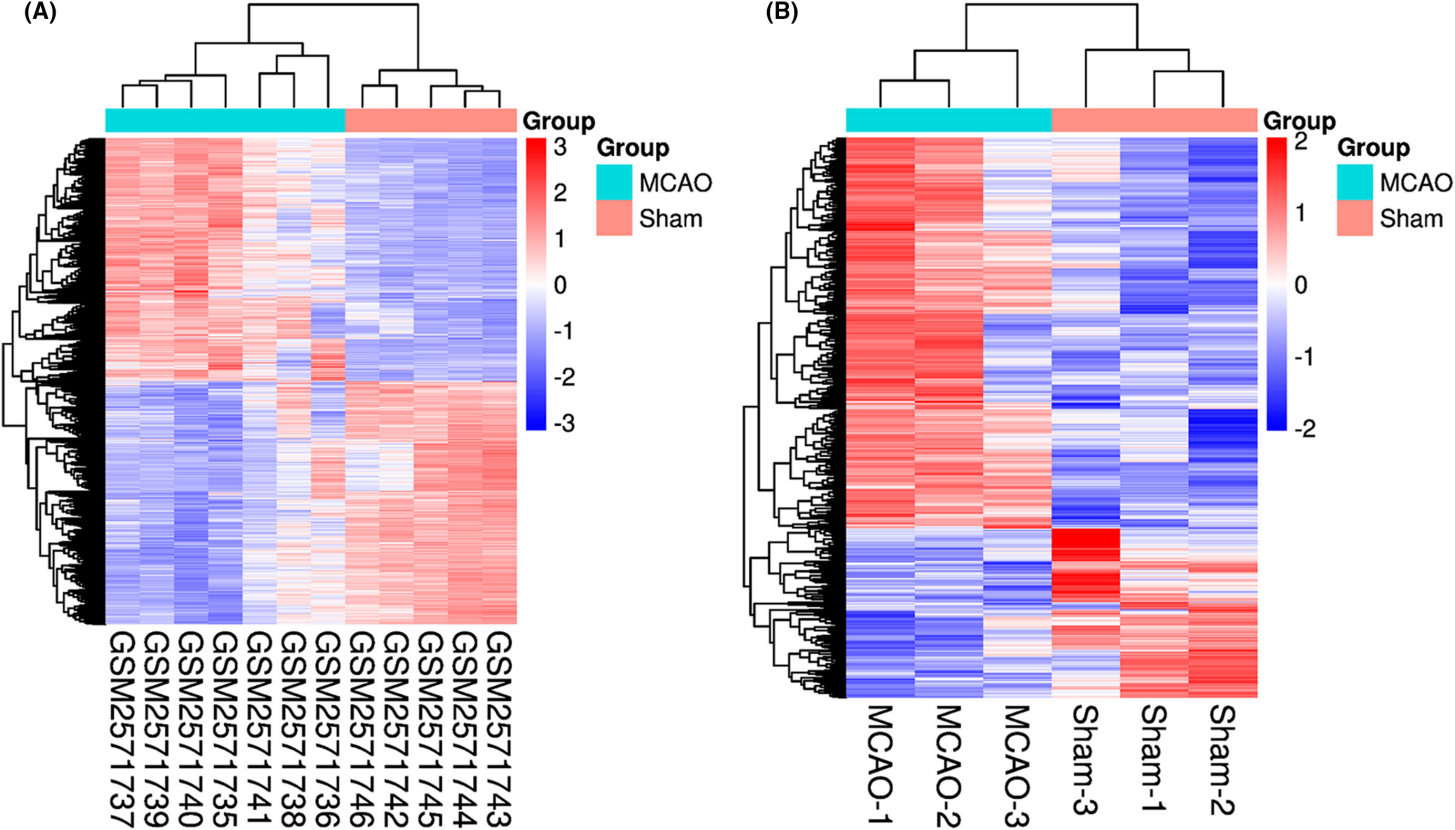
Identification of MCAO/R‐related DEGs in GSE97537 and GSE160500 datasets. (A) Heat map of MCAO/R‐related expression gene based on GSE97537 dataset; (B) Heat map of MCAO/R‐related expression gene based on GSE160500 dataset.

**FIGURE 2:**
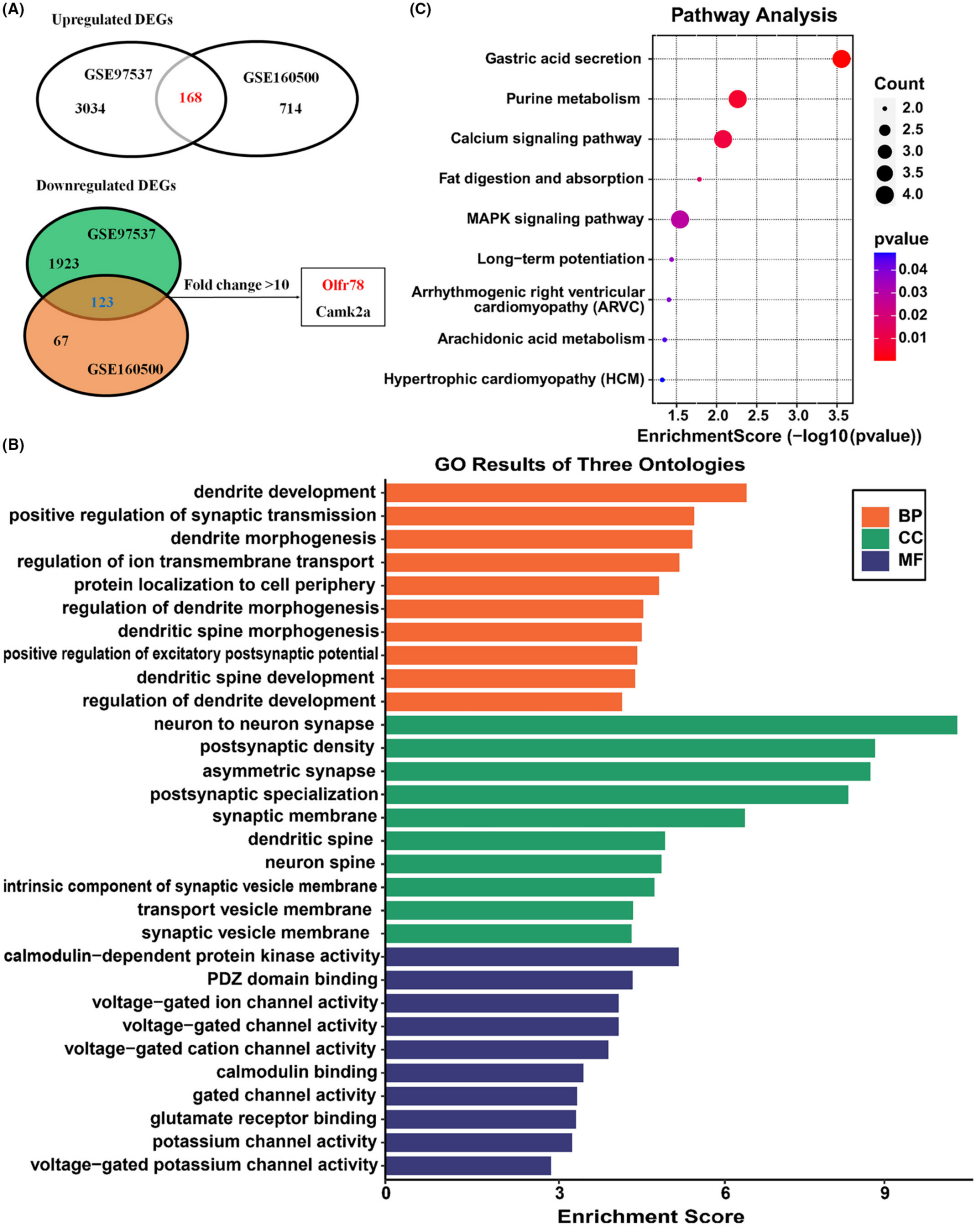
Identification and enrichment analysis of overlapping differentially expressed genes (DEGs) in GSE97537 and GSE160500 datasets. (A) Identification of overlapping DEGs in GSE97537 and GSE160500 datasets, and Olfr78 and Camk2a were screened as the Hub gene; (B) Gene ontology (GO) analysis revealed major enrichment of downregulated MCAO/R‐related DEGs in biological processes (BPs), cellular components (CCs), and molecular functions (MFs); (C) KEGG analysis revealed main enrichment of downregulated MCAO/R‐related DEGs in the signalling pathways (*p*‐value decreases as the colour tends to red. The larger the circle, the more genes are enriched).

Two datasets in the GEO database were screened to find important regulatory genes of ischemic stroke. The heatmap showed that the expression levels of genes in these two datasets were significantly different between the sham and MCAO/R groups (Figure 1A,B). According to the standard adjusted *p*‐value < 0.05 and |logFc| > 1.2, 5248 DEGs were screened from the GSE97537 dataset, of which 3202 were upregulated and 2046 were downregulated (Figure 2A). GO functional enrichment and KEGG pathway enrichment analyses by using the clusterProfiler R package showed that MCAO/R‐related DEGs were mainly related to biological functions such as ribonucleoprotein complex biogenesis, cytosolic ribosome, synaptic membrane, and structural constituent of ribosome. Moreover, MCAO/R‐related DEGs enriched in Ribosome, Calcium signalling pathway, and MAPK signalling pathway (Figure S1A–D). Based on the analysis of GSE160500 dataset, 1072 MCAO/R‐related DEGs were obtained (Figure 2A), which were mainly related to the functions of chromosome segregation, neuron to neuron synapse, voltage‐gated ion channel activity, voltage‐gated channel activity, Cell adhesion molecules (CAMs), MAPK signalling pathway, Calcium signalling pathway, and other pathways (Figure S2A–D). Meanwhile, 291 overlapping MCAO/R‐related DEGs were screened from the GSE97573 and GSE160500 datasets by joint analysis, of which 168 were overlapping upregulated DEGs and 123 were overlapping downregulated DEGs (Figure 2A). GO functional enrichment and KEGG pathway enrichment analyses showed that overlapping DEGs were mainly related to dendrite development, positive regulation of synaptic transmission, neuron to neuron synapse, extracellular matrix, calcium‐dependent protein binding, voltage‐gated ion channel activity, Calcium signalling pathway, and MAPK signalling pathway (Figures 2B,C and S3A,B). Finally, Olfr78 and CAmk2a were identified as hub genes by screening DEGs that were downregulated more than 10‐fold (Figure 2A). Since the role of Olfr78 in CIRI is unclear, we used Olfr78 as a target gene for subsequent studies.

### 3.2 The expression of Olfr78 was downregulated in MCAO/R rats and OGD/R‐stimulated neurons

**FIGURE 3:**
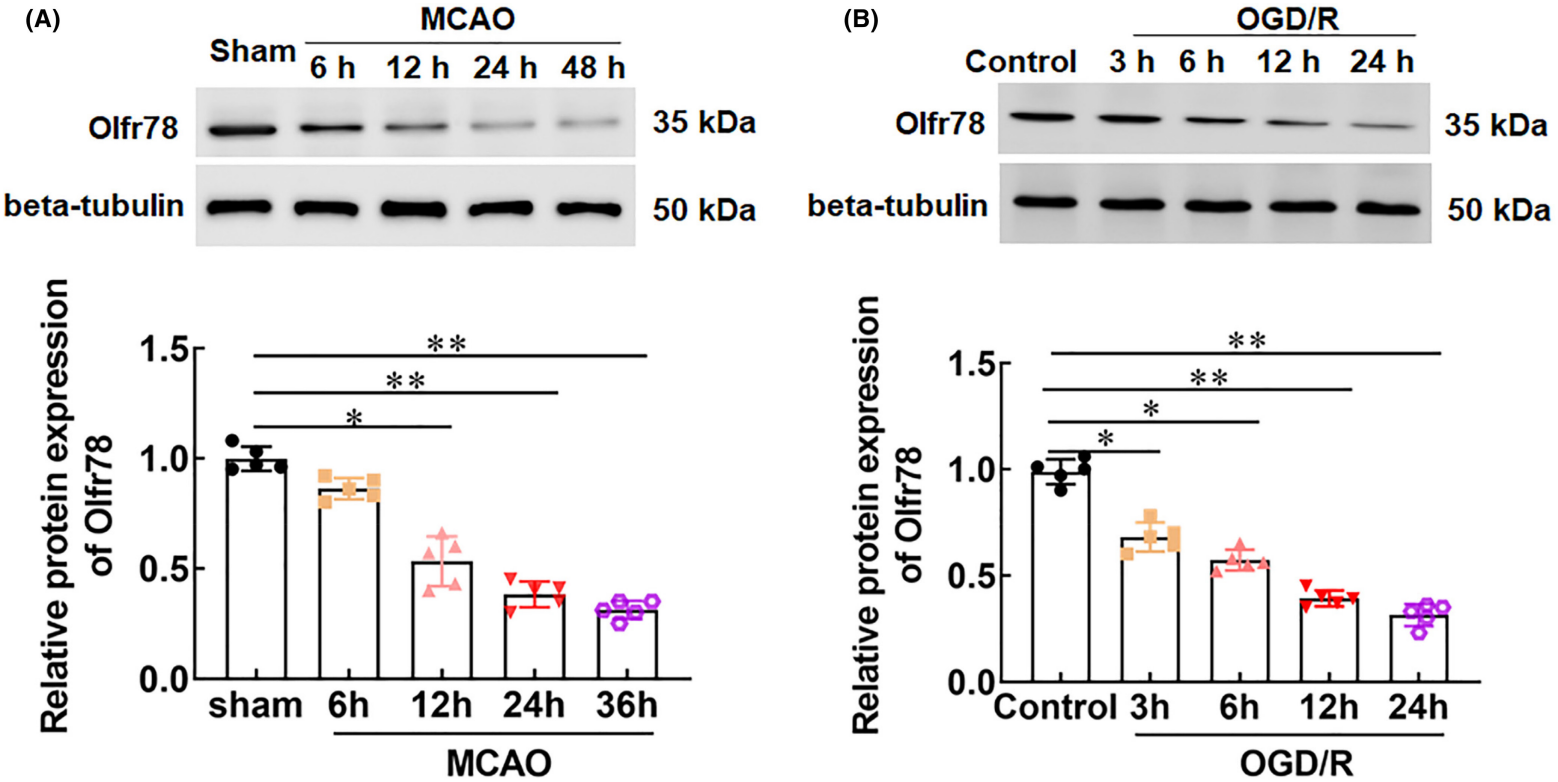
The expression of Olfr78 in middle cerebral artery occlusion/reperfusion (MCAO/R) rats and oxygen‐glucose deprivation/reoxygenation (OGD/R)‐stimulated neurons. (A) A rat model of MCAO/R was constructed, and the expression of Olfr78 in brain tissues of MCAO/R rats was detected with RT‐qPCR at 6, 12, 24, and 48 h after surgery, *n* = 10. (B) An in vitro model of OGD/R‐stimulated neuronal cells was constructed, and the expression of Olfr78 was detected with RT‐qPCR at 3, 6, 12, and 24 h after modelling, *n* = 5. Data are expressed as means ± SEM. Statistical differences were evaluated by using one‐way ANOV, and followed by Tukey HSD test. Compare with the sham group or control group, *p < 0.05, **p < 0.01.

As shown in Figure 3C, Olfr78 and Camk2a were selected as hub genes from 123 overlapping downregulated DEGs, while the role of Olfr78 in CIRI is unclear. Therefore, we selected Olfr78 as the target gene for subsequent studies. To investigate the role of Olfr78 in CIRI, we constructed an in vivo rat model of MCAO/R, and found that the expression of Olfr78 in brain tissues of MCAO/R rats was decreased from 12 h after surgery, in a time‐dependent manner (Figure 3A). Furthermore, we also constructed an in vitro model of ischaemia–reperfusion by using OGD/R‐induced neuronal cells, and the results showed that after 3 h of modelling, the expression of Olfr78 was markedly decreased in a time‐dependent manner (Figure 3B).

### 3.3 Overexpressing Olfr78 inhibits Ca^2+^ deposition and OGD/R‐induced neuronal apoptosis

**FIGURE 4:**
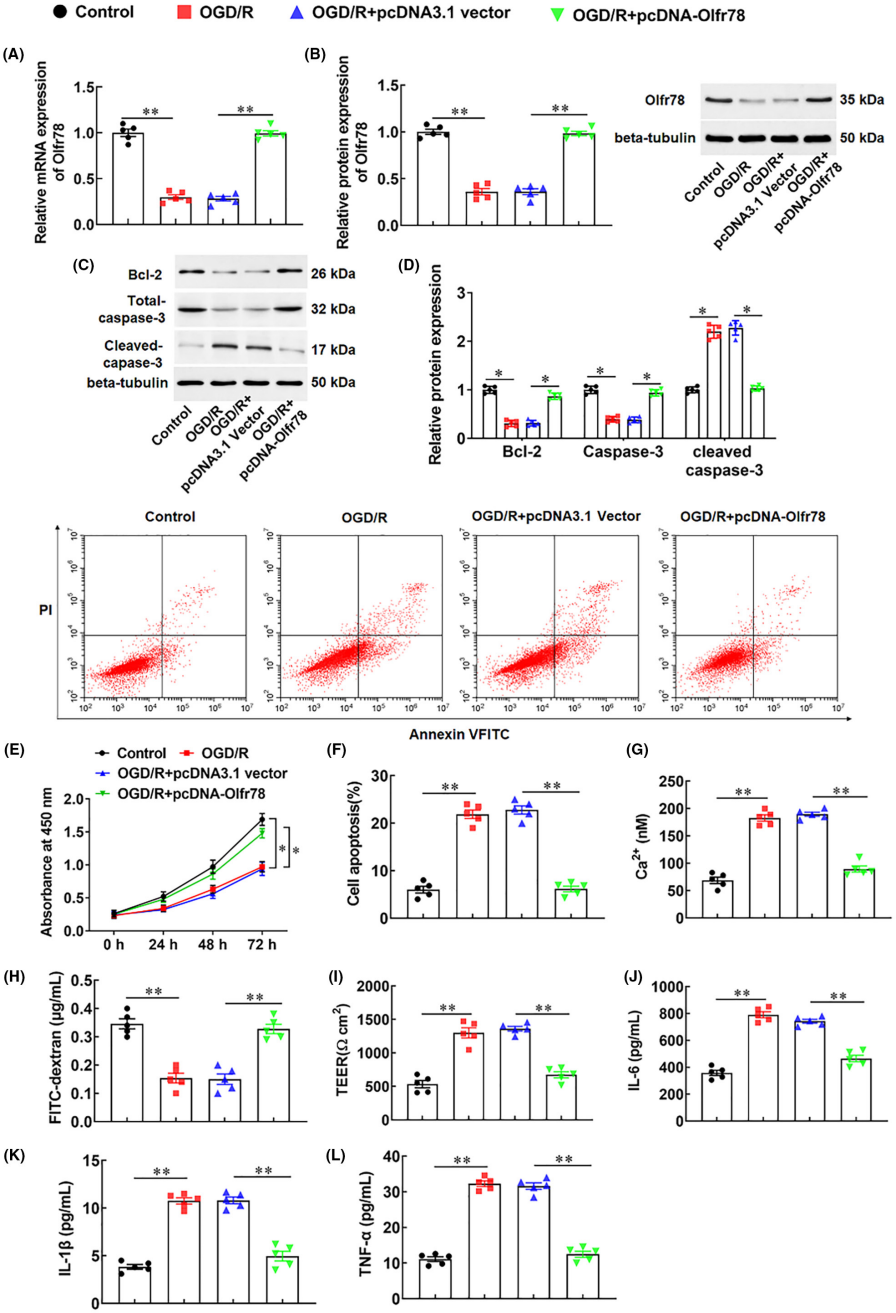
Effects of Olfr78 overexpression on Ca2+ deposition and neuronal apoptosis induced by oxygen‐glucose deprivation/reoxygenation (OGD/R). Neurons were transfected with Olfr78 overexpression vector (pcDNA‐Olfr78) or empty vector (pcDNA3.1 vector), and then treated with OGD/R for 12 h. (A) The expression of Olfr78 was detected with RT‐qPCR. (B–D) The protein levels of Olfr78 (B), BCL‐2, Total‐caspase‐3, and Cleaved‐caspase‐3 (D) were detected with Western blotting. (E) Cell viability was detected with CCK‐8. (F) Cell apoptosis was detected with the flow cytometry. (G) The transcellular resistance (TEER) of neurons was measured. (H) The permeability of cells was measured by using FITC‐dextran permeability assay. (I) The concentration of Ca^2+^ was measured by colorimetry. (J–L) The secretion of inflammatory factors IL‐6 (J), IL‐1β (K), and TNF‐α (L) was detected with ELISA. Data are expressed as means ± SEM, *n* = 5. Statistical differences were evaluated by using one‐way and two‐way ANOV, and followed by Tukey HSD test. Compare with the control group or the OGD/R + pcDNA3.1 vector group, *p < 0.05, **p < 0.01.

To explore the role of Olfr78 in CIRI, we transfected Olfr78 overexpression vector (pcDNA‐Olfr78) or empty vector (pcDNA3.1 vector) into neuronal cells. The results showed that the expression levels of Olfr78 mRNA and protein in neuron were significantly decreased after OGD/R treatment, while the expression level of Olfr78 was significantly increased in neurons transfected with pcDNA‐Olfr78 (Figure 4A,B), indicating the successful construction of Olfr78 overexpression vector. Next, cell viability was tested and found that OGD/R treatment significantly reduced neuronal viability, while overexpression of Olfr78 increased neuronal viability (Figure 4E). At the same time, we observed that OGD/R treatment significantly induced neuronal apoptosis, while overexpression of Olfr78 inhibited cell apoptosis, reduced the expression of apoptosis related protein Ceaved‐caspase‐3, and promoted the expression of anti‐apoptotic protein BCL‐2 (Figure 4C,D,F). Furthermore, we also evaluated the barrier integrity of neuronal cells by measuring transcellular resistance (TEER), the permeability of cells by FITC‐dextran permeability assay, and the concentration of Ca^2+^ in neurons by colorimetry. The results showed that after OGD/R treatment, the barrier integrity of neuronal cells was decreased, cell permeability was increased, and the concentration of Ca^2+^ was increased, while overexpression of Olfr78 inhibited OGD/R‐induced barrier dysfunction and Ca^2+^ deposition in neurons (Figure 4G–I). In addition, we also found that OGD/R treatment significantly promoted the secretion of inflammatory factors IL‐6, IL‐1β, and TNF‐α in neurons, while overexpression of Olfr78 inhibited the secretion of inflammatory factors (Figure 4J–L). Meanwhile, OGD/R treatment significantly promoted the secretion of IL‐18 and increased the protein levels of NLRP3 and Cleaved‐caspase‐1 in neurons, while overexpression of Olfr78 inhibited the activation of NLRP3 inflammasome in neurons (Figure S4A–D). The above results suggest that overexpression of Olfr78 reduces inflammation response by inhibiting Ca^2+^ deposition, thereby inhibiting OGD/R‐induced neuronal apoptosis.

### 3.4 Olfr78 interacts with Prkaca and promotes Prkaca expression

**FIGURE 5:**
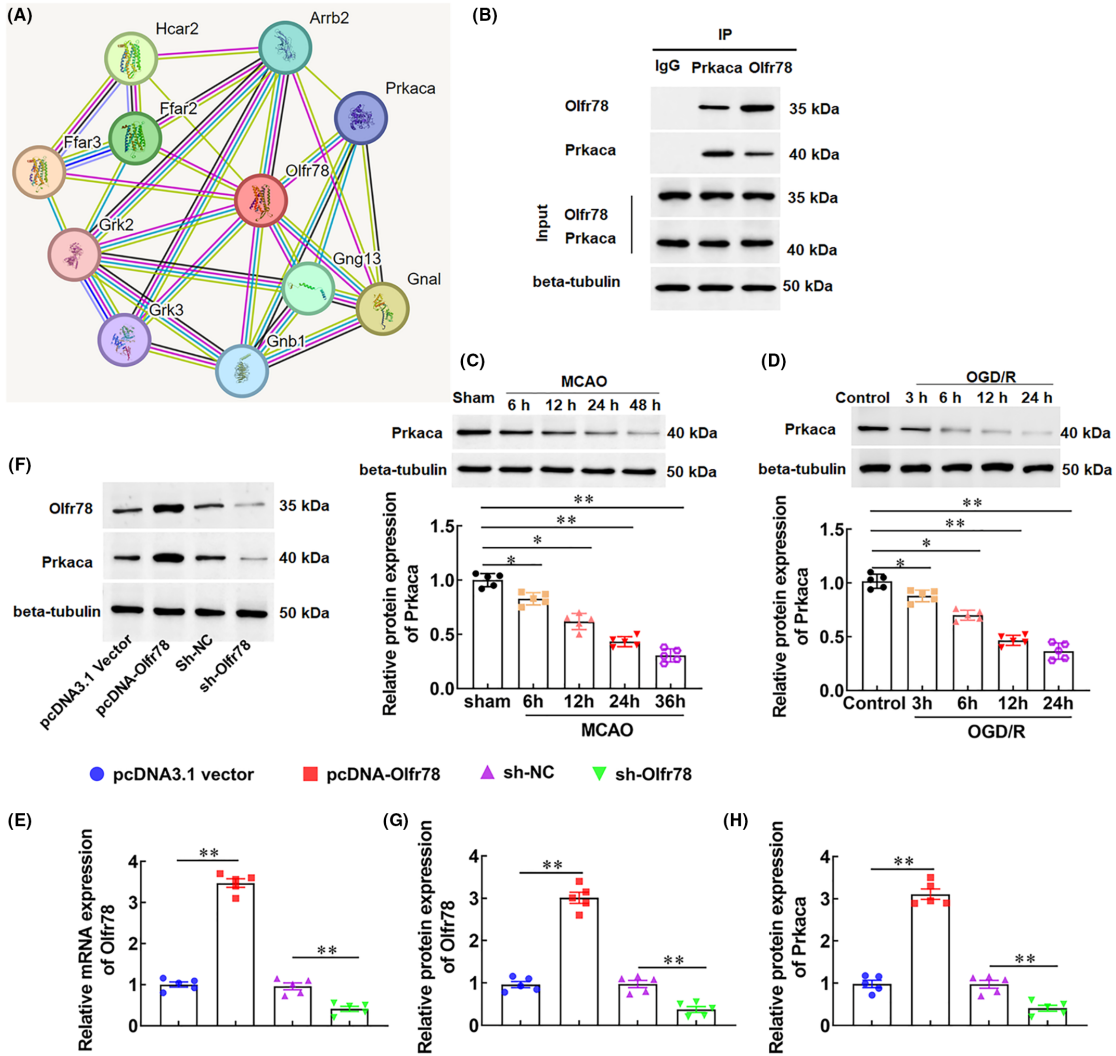
Olfr78 interacts with Prkaca and promotes Prkaca protein expression. (A) The STRING online database (https://cn.string‐db.org/) was used to predict the interaction between Olfr78 protein and Prkaca protein. (B) The interaction between Olfr78 protein and Prkaca protein was validated by Co‐IP analysis. (C) The protein level of Prkaca in brain tissues of rats in the sham group and the middle cerebral artery occlusion/reperfusion (MCAO/R) group was detected with Western blotting at 6, 12, 24, and 48 h after surgery, *n* = 10. (D) The protein level of Prkaca in neurons after oxygen‐glucose deprivation/reoxygenation (OGD/R) treatment for 3, 6, 12, and 24 h was detected with Western blotting. Neurons were transfected with Olfr78 overexpression vector (pcDNA‐Olfr78) or shRNA (sh‐Olfr78) or their control vector, and then treated with OGD/R for 12 h. (E) The mRNA expression of Olfr78 was detected with RT‐qPCR. (F–H) The protein levels of Olfr78 (G) and Prkaca (H) were detected with Western blotting. Data are expressed as means ± SEM, *n* = 5. Statistical differences were evaluated by using one‐way ANOV, and followed by Tukey HSD test. Compare with the sham group, control group, pcDNA3.1 vector or sh‐NC, *p < 0.05, **p < 0.01.

To explore the regulatory mechanism of Olfr78 in CIRI, the STRING online database (https://cn.string‐db.org/) was used to predict the interaction protein of Olfr78 and found that Olfr78 protein may be interact with Prkaca protein (Figure 5A). Next, the interaction between Olfr78 protein and Prkaca protein was validated by Co‐IP analysis (Figure 5B). Moreover, we detected the expression level of Prkaca in MCAO/R model rats and found that compared with the sham group, the protein level of Prkaca in brain tissues of MCAO/R model rats showed a time‐dependent decrease after surgery (Figure 5C). We also observed a significant decrease in Prkaca protein levels in OGD/R treated neurons in a time‐dependent manner (Figure 5D). In addition, overexpression of Olfr78 significantly promoted Prkaca protein expression, while silencing Olfr78 inhibited Prkaca protein expression (Figure 5E–H). These results suggest that Olfr78 interacted with Prkaca and promoted Prkaca protein expression.

### 3.5 Olfr78 inhibits OGD/R‐induced Ca^2+^ deposition and neuronal apoptosis by upregulating Prkaca expression

**FIGURE 6:**
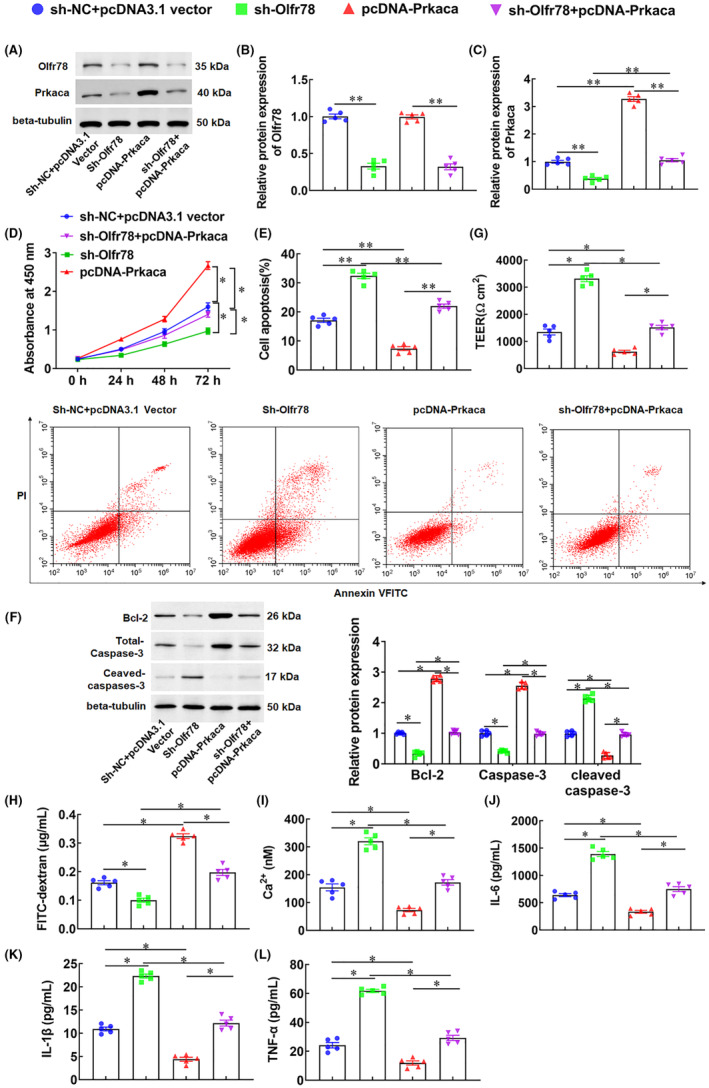
Olfr78 inhibits oxygen‐glucose deprivation/reoxygenation (OGD/R)‐induced Ca^2+^ deposition and neuronal apoptosis by upregulating Prkaca protein expression. Neurons were transfected with sh‐Olfr78 or co‐transfected with Prkaca overexpression vector (pcDNA‐Prkaca). (A–C) The protein levels of Olfr78 (B) and Prkaca (C) were detected with Western blotting. (D) Cell viability was detected with CCK‐8. (E) Cell apoptosis was detected with the flow cytometry. (F) The protein levels of BCL‐2, Total‐caspase‐3, and Cleaved‐caspase‐3 were detected with Western blotting. (G) The transcellular resistance (TEER) of neurons was measured. (H) The permeability of cells was measured by using FITC‐dextran permeability assay. (I) The concentration of Ca^2+^ was measured by colorimetry. (J–L) The secretion of inflammatory factors IL‐6 (J), IL‐1β (K), and TNF‐α (L) was detected with ELISA. Data are expressed as means ± SEM, *n* = 5. Statistical differences were evaluated by using one‐way and two‐way ANOV, and followed by Tukey HSD test. Compare with the sh‐NC + pcDNA3.1 vector group, sh‐Olfr78 group, or the pcDNA‐Prkaca group, *p < 0.05, **p < 0.01.

Next, we investigate whether Prkaca plays a role in CIRI. Neuronal cells were transfected with sh‐Olfr78 or co‐transfected with Prkaca overexpression vector (pcDNA‐Prkaca). The results showed that silencing Olfr78 significantly reduced the protein levels of Olfr78 and Prkaca, while overexpression of Prkaca increased the protein level of Prkaca (Figure 6A–C). Silencing Olfr78 inhibited neuronal viability, overexpression of Prkaca promoted neuronal viability, while co‐transfection with sh‐Olfr78 and pcDNA‐Prtaca reversed the inhibitory effect of sh‐Olfr78 on neuronal viability (Figure 6D). Furthermore, silencing Olfr78 significantly induced neuronal apoptosis, promoted cleaved‐caspase‐3 expression, and inhibited BCL‐2 expression, while overexpression of Prkaca counteracted the induction effect of Olfr78 silencing on cell apoptosis (Figure 6E,F). Moreover, silencing Olfr78 decreased the barrier integrity of neurons, enhanced cell permeability, and promoted Ca^2+^ deposition and the secretion of inflammatory factors IL‐6, IL‐1β, while overexpression of Prkaca counteracted effects of Olfr78 silencing on neurons (Figure 6G–L). The above results indicate that overexpressing Olfr78 inhibited neuronal apoptosis by upregulating Prkaca expression.

### 3.6 Overexpression of Prkaca inhibits OGD/R‐induced neuronal apoptosis by activating the cAMP/PKA‐MAPK pathway

**FIGURE 7:**
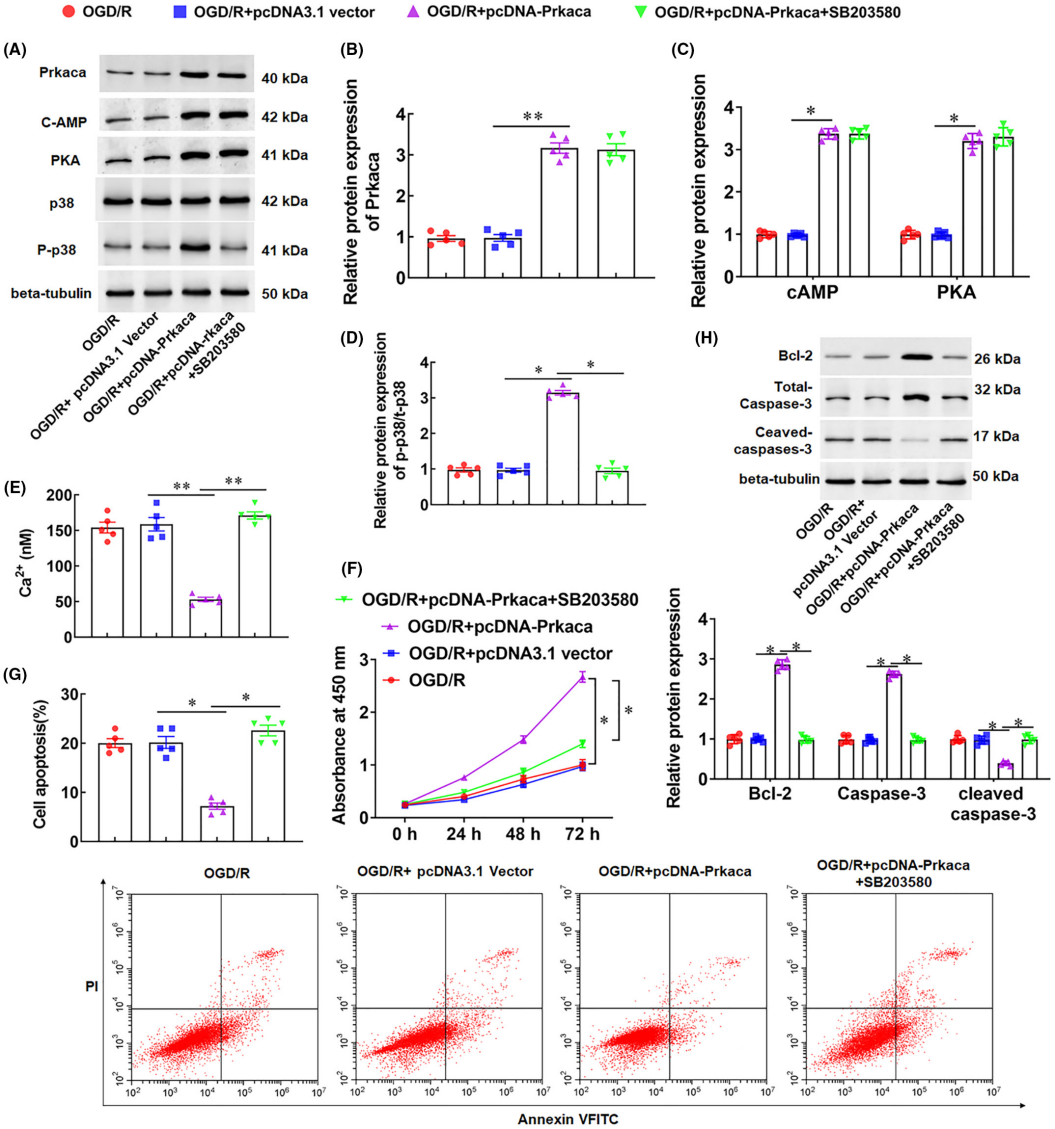
Overexpression of Prkaca inhibits oxygen‐glucose deprivation/reoxygenation (OGD/R)‐induced neuronal apoptosis through the cAMP/PKA‐MAPK pathway. Neurons were transfected with Prkaca overexpression vector (pcDNA‐Prkaca) or co‐transfected with SB203580, a p38 inhibitor. (A–D) The protein levels of Prkaca (B), Camp, PKA and p‐p38 (D) were detected with Western blotting. (E) The concentration of Ca^2+^ was measured by colorimetry. (F) Cell viability was detected with CCK‐8. (G) Cell apoptosis was detected with the flow cytometry. (H) The protein levels of BCL‐2, Total‐caspase‐3, and Cleaved‐caspase‐3 were detected with Western blotting. Data are expressed as means ± SEM, *n* = 5. Statistical differences were evaluated by using one‐way and two‐way ANOV, and followed by Tukey HSD test. Compare with the OGD/R + pcDNA3.1 vector group, or the pcDNA‐Prkaca group, *p < 0.05, **p < 0.01.

KEGG analysis showed that downregulated genes in MCO/R were significantly enriched not only in the calcium signalling pathway, but also in the MAPK signalling pathway. Early reports showed that a signal‐transduction cascade from cAMP/PKA to MAPK is neuron‐specific.^36^, ^37^ Therefore, we speculated that Prkaca inhibited OGD/R‐induced neuronal apoptosis through the cAMP/PKA‐MAPK pathway. To verify our hypothesis, neuronal cells were transfected with Prkaca overexpression vector (pcDNA‐Prkaca) or co‐transfected with SB203580, a p38 inhibitor. The results showed that overexpression of Prkaca significantly promoted the protein expression of cAMP and PKA, and increased the phosphorylation level of p38 (Figure 7A–D). Moreover, overexpression of Prkaca significantly reduced Ca^2+^ deposition in neuronal cells treated with OGD/R (Figure 7E). Meanwhiles, overexpression of Prkaca significantly promoted neuronal viability, inhibited OGD/R‐induced neuronal apoptosis, promoted cleaved‐caspase‐3 expression, and inhibited BCL‐2 expression (Figure 7F–H). However, SB203580, a p38 inhibitor, counteracted the effect of Prkaca overexpression on neurons (Figure 7E–H). The above results suggest that Prkaca inhibited OGD/R‐induced neuronal apoptosis by activating the cAMP/PKA‐MAPK pathway.

### 3.7 Overexpression of Olfr78 or Prkaca alleviates brain injury in MCAO/R rats

**FIGURE 8:**
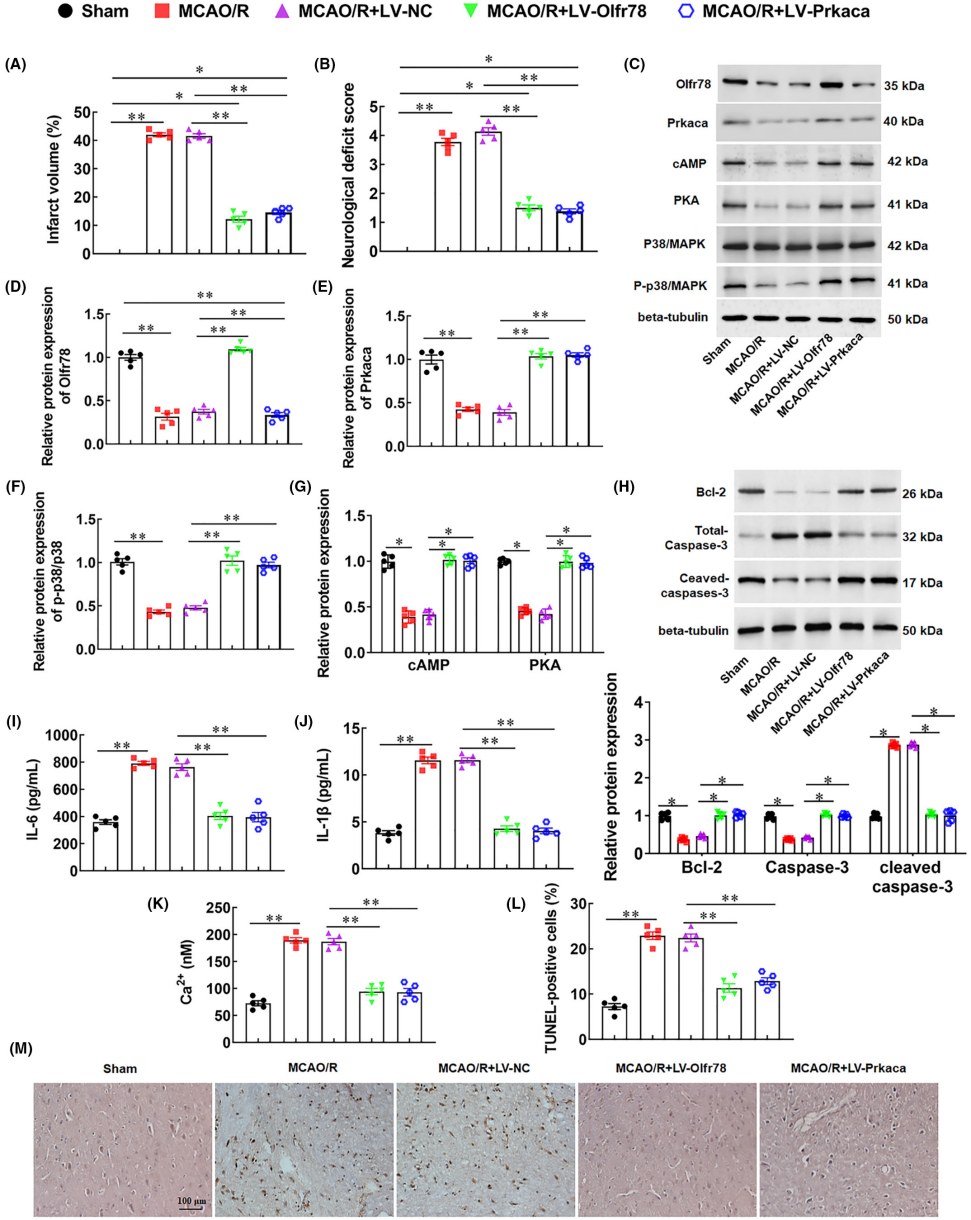
Overexpression of Olfr78 or Prkaca alleviates brain injury in MCAO/R rats. Forty SD rats were randomly divided into five groups: the sham group, MCAO/R model group, MCAO/R + LV‐NC group, MCAO/R + LV‐Olfr78 group, and MCAO/R + LV‐Prkaca group, with 8 rats in each group. (A) Statistics of infarct volume of brain tissues of rats in each group; (B) Statistics of neurological deficit scores of rats in each group; (C–H) The protein levels of Olfr78 (D), Prkaca (E), p38, p‐p38 (F), Camp, PKA (G), BCL‐2, Total‐caspase‐3, and Cleaved‐caspase‐3 (H) in brain tissues of rats were measured by Western blotting; (I, J) The concentration of IL‐6 (I) and IL‐1β (J) in serum of rats in each group was detected with ELISA; (K) The concentration of Ca^2+^ was measured by colorimetry. (L, M) Neuronal apoptosis in brain tissues of rats in each group was detected with TUNEL staining. Data are expressed as means ± SEM, *n* = 5. Statistical differences were evaluated by using one‐way, and followed by Tukey HSD test. Compare with the sham group, MCAO/R group, or the MCAO/R + LV‐NC group, *p < 0.05, **p < 0.01.

TTC staining results showed that the brain tissues were ruddy and the cerebral infarction area was pale, and the infarct volume significantly increased in MCAO/R model rats, while the infarct volume of the brain tissues was significantly reduced in rats of the MACO+LV‐Olfr78 group and MACO+LV‐Prkaca group (Figures 8A and S5). Furthermore, compared to the sham group, MCAO/R model rats showed a significant increase in neurological deficit scores, while overexpression of Olfr78 or Prkaca markedly decreased scores in MACO/R rats (Figure 8B). The levels of Olfr78, Prkaca, cAMP, and PKA proteins in brain tissues of MCAO/R model rats were significantly reduced, and the phosphorylation level of p38 was also significantly reduced. Overexpression of Olfr78 or Prkaca promoted the expression of cAMP and PKA proteins, as well as the phosphorylation of p38 (Figure 8C–G). Meanwhiles, Overexpression of Olfr78 or Prkaca increased the expression level of anti‐apoptotic protein Bcl‐2 and decreased the protein level of Pro‐apoptotic protein cleaved‐caspase‐3 (Figure 8H). In addition, compared with the rats in the sham group, the concentration of IL‐6, IL‐1β, and IL‐18 was elevated, the protein levels of NLRP3 and Cleaved‐caspase‐1 were upregulated, and Ca^2+^ deposition and the number of cell apoptotic were significantly increased in the MCAO/R model rats, while overexpression of Olfr78 or Prkaca significantly decreased the concentration of IL‐6, IL‐1β, and IL‐18, inhibited the protein expression of NLRP3 and Cleaved‐caspase‐1, reduced Ca^2+^ deposition and inhibited neuronal apoptosis (Figures 8I–M and S6A,B). To further verify the role of Olfr78 and Prkaca in CIRI, we treated MCAO/R model rats with propionate, an activator of Olfr78, and DS89002333, an inhibitor of Prkaca. The results showed that propionate markedly reduced the infarct volume of brain tissues and the neurological deficit scores, decreased the concentration of inflammatory factors IL‐6 and IL‐1β, and inhibited neuronal apoptosis, while DS89002333 counteracted the neuroprotective effect of propionate on MCAO/R rats (Figure S7A–F). The above results show that overexpression of Olfr78 or Prkaca significantly alleviated brain injury in MCAO/R rats by reducing Ca^2+^ deposition to inhibit neuronal apoptosis.

In addition, we also evaluated the relationship between the expression levels of Olfr78 and Prkaca and the clinical characteristics of patients with acute ischemic stroke. As shown in Table 1, hypertension, smoking, drinking, or diabetes was more prevalent among acute ischemic stroke patients with Olfr78 low expression than acute ischemic stroke patients with Olfr78 high expression (*p* < 0.05). The serum levels of TC, TG, and LDL‐C in the acute ischemic stroke patients with Olfr78 low expression was also higher than those in the acute ischemic stroke patients with Olfr78 high expression (*p* < 0.05). Conversely, the level of HDL‐C in serum of the acute ischemic stroke patients with Olfr78 low expression was significantly lower than those in the acute ischemic stroke patients with Olfr78 high expression (*p* < 0.05). In terms of gender and age, there was no significant difference between ischemic stroke patients with low and high expression of Olfr78 (all *p* > 0.05). Meanwhile, compared with the acute ischemic stroke patients with Prkaca high expression, hypertension, smoking, or diabetes was more prevalent in the acute ischemic stroke patients with Prkaca low expression (*p* < 0.05). The serum levels of TC, TG, and LDL‐C in the acute ischemic stroke patients with Prkaca low expression was also higher than the acute ischemic stroke patients with Prkaca high expression (*p* < 0.01). The serum level of HDL‐C in the acute ischemic stroke patients with Prkaca low expression was significantly lower than the acute ischemic stroke patients with Prkaca high expression (*p* < 0.001). In terms of gender and age was not any significant difference between ischemic stroke patients with Prkaca low expression and Prkaca high expression (all *p* > 0.05, Table 2). In summary, we determined that Olfr78 and Prkaca may be key genes that play important roles in the pathogenesis of CIRI.

**TABLE 1:** The relationship between OR51E2 (Olfr78) expression and clinicopathologic factors of acute ischemic stroke patients.

Characteristics	Cases (*n* = 124)	OR51E2 expression		F/*χ* ^2^	*p*‐value
		Low (*n* = 63)	High (*n* = 61)		
Age (years)		53.0 ± 9.4	61.0 ± 10.7	1.476	0.465
Gender (%)				0.342	0.559
Male	74	36 (57.1%)	38 (62.3%)		
Female	50	27 (42.9%)	23 (37.7%)		
Smoker	28	21 (42.9%)	7 (11.5%)	8.470	0.004
Drinker	22	17 (27.0%)	5 (8.2%)	7.496	0.006
Hypertension	39	25 (39.7%)	14 (23.0%)	4.068	0.044
Diabetes mellitus	24	16 (25.4%)	8 (13.1%)	2.995	0.084
TG (mmol/L)		1.91 ± 0.21	1.15 ± 0.12	11.451	0.042
TC (mmol/L)		2.13 ± 0.17	0.97 ± 0.09	0.872	<0.001
HDL‐C (mmol/L)		0.37 ± 0.55	0.93 ± 0.51	6.405	0.031
LDL‐C (mmol/L)		2.33 ± 0.41	0.72 ± 0.25	1.013	<0.001
NIHSS scores		4.43 ± 0.75	2.13 ± 0.74	3.152	0.005

**TABLE 2:** The relationship between Prkaca expression and clinicopathologic factors of acute ischemic stroke patients.

Characteristics	Cases (*n* = 124)	Olfr78 expression		F/*χ* ^2^	*p*‐value
		Low (*n* = 62)	High (*n* = 62)		
Age (years)		55.12 ± 10.6	58.76 ± 10.7	3.875	0.884
Gender (%)				0.033	0.857
Male	69	35 (56.5%)	34 (54.8%)		
Female	55	27 (43.5%)	28 (45.2%)		
Smoker	28	19 (30.6%)	8 (12.9%)	5.729	0.017
Drinker	22	11 (17.7%)	5 (8.1%)	2.583	0.108
Hypertension	39	27 (43.5%)	13 (21.0%)	7.233	0.007
Diabetes mellitus	24	17 (27.4%)	8 (12.9%)	4.058	0.044
TG (mmol/L)		1.87 ± 0.32	1.08 ± 0.14	1.873	0.002
TC (mmol/L)		2.33 ± 0.24	1.03 ± 0.12	6.541	<0.001
HDL‐C (mmol/L)		0.32 ± 0.18	0.97 ± 0.25	0.379	<0.001
LDL‐C (mmol/L)		2.18 ± 0.51	0.66 ± 0.34	0.975	<0.001
NIHSS scores		4.35 ± 0.67	2.28 ± 0.71	2.135	<0.001

## 4 DISCUSSION

Ischemic stroke causes high mortality and disability rates worldwide, so developing effective treatment methods is crucial for it.^1^, ^38^ At present, the main treatment method for stroke is mechanical thrombectomy (MT). Although MT significantly improves the success rate of recanalization after acute occlusion of the internal carotid artery or middle cerebral artery and improves the sequelae of neurological injury, it leads to neuronal cell injury and death, resulting in secondary injury to the body.^2^, ^4^, ^39^ Many factors affect CIRI, and no specific treatment is currently available. Therefore, it's of great significance to explore the mechanism of CIRI and find new prevention and therapeutic strategies.

With the advancement of biological technology, the development of next‐generation sequencing technology has made it possible to detect and analyse genes with low or high expression in certain cell types or diseases.^40^, ^41^ In this study, Olfr78 was obtained as a central gene, which was found to be involved in the regulation of neuronal apoptosis after cerebral ischemia through the screening and enrichment analysis of gene expression profiles of rat cerebral ischemia related datasets. We combined bioinformatics and animal experiments to further confirm overlapping downregulated DEGs Olfr78, which was downregulated in a time‐dependent manner after CIRI. Overexpression of Olfr78 reduced Ca^2+^ overload, inhibited neuronal apoptosis induced by OGD/R in vitro and improved brain injury in MCAO/R rats in vivo. Our results may provide a new direction for elucidating the molecular mechanism of neuronal apoptosis after stroke.

The G protein coupled receptors (GPCRs) are essential for many organs of the body to function, including visual and olfactory signal transduction, emotional and behavioural regulation, nervous and immune systems, inflammation, and many other biological processes.^40^, ^42^ Olfr78, a member of the GPCR family, mediates olfactory chemosensation in the nose, and has been identified the mainly receptor for Short‐chain fatty acids (SCFAs).^9^ Interestingly, Olfr78 uses several different names according to the species and organ types analysed.^12^, ^42^ Olfr78 was first found to be highly expressed in human prostate, so it was originally named prostate‐specific G protein coupled receptor (PSGR),^16^ and Olfr78 is known as Olr59 in rats^43^ and OR51E2 in humans.^44^ Previous reports have shown that Olfr78 is involved in the regulation of blood pressure in the kidney,^16^, ^22^ while the high expression of Olfr78 in the prostate is involved in the development of cancer.^16^ Furthermore, Olfr78 in arteriolar smooth muscle cells is not only involved in the regulation of blood pressure, but also in the fine‐tuning of blood supply by regulating vascular calcification, thus participating in the disease progression of chronic kidney injury and atherosclerosis.^17^, ^18^, ^19^ Meanwhiles, a report showed that Olfr78 regulates cardiorespiratory responses of erythrocytes to hypobaric hypoxia, and Olfr78 knockout mice have a significant loss of cardiopulmonary adaptation to hypobaric hypoxia, as well as a significant loss of carotid body sensory nerve response.^45^ Furthermore, Olfr78 has been reported to be an integral component of the hypoxic sensing mechanism of the carotid body to a wide range of hypoxia levels.^13^ A report showed that redox modification of Olfr78 participates in carotid body activation by hypoxia to regulate breathing.^46^ The results consistent with the above reports were observed in our study, the expression of Olfr78 was significantly decreased after hypoxic injury. In addition, some studies have shown that the release of inflammatory factors caused by NLRP3 inflammasome activation is a significant pathological feature of cardiovascular disease and brain injury.^47^, ^48^, ^49^ In our study, the levels of inflammatory factors were significantly increased in OGD/R‐induced neurons and MCAO/R rats, and overexpression of Olfr78 reduced secretion of inflammatory factors, inhibited the activation of NLRP3 inflammasome and neuronal apoptosis in OGD/R‐induced neurons, and alleviated brain injury of MCAO/R rats in vivo.

To further explore the role of Olfr78 in CIRI, we also predicted the interacting protein of Olfr78, Prkaca, through bioinformatics online database, and validated the interaction between Olfr78 protein and Prkaca protein by Co‐IP analysis. Prkaca gene encodes the Cα catalytic subunit of protein kinase A (PKA‐Cα). PKA, also known as cAMP‐dependent protein kinase A, is involved in signal transduction of cell apoptosis.^23^, ^24^ cAMP induces PKA activation, mediates apoptosis target genes Bcl‐2 and Bax expression, and promotes cell apoptosis, in which intracellular calcium signalling plays a crucial role.^23^, ^50^, ^51^ Various stimuli lead to calcium overload, further leading to the release of cytochrome C and apoptosis inducing factors.^51^, ^52^ The pathophysiological process of cerebral ischemia involves multiple potential mechanisms, including excitotoxicity, ion imbalance, oxidative stress, inflammation, and cell apoptosis.^2^, ^5^ Calcium overload plays a crucial role in the process of CIRI and triggers ischemic cascade reactions, which leads to irreversible neuronal damage.^53^, ^54^ Previous studies have shown that the occurrence of neurological diseases is related to intracellular calcium homeostasis disorders.^50^, ^55^ PKA plays a crucial role in the calcium signalling pathway of neurological diseases, and the activation of cAMP/PKA signalling pathway in the striatum is highly correlated with the loss of dopaminergic neurons in PD rats.^56^, ^57^ At present, inhibiting Ca^2+^ signalling transmission mediated Ca^2+^ overload is considered as a promising method for combating CIRI.^58^, ^59^ Moreover, a report has showed that the expression of Prkaca enhances the intensity of the PKA‐RCA1 signal transmission, which contributes to the development of neurons and the formation of dendritic spines.^60^ Meanwhile, activation of the cAMP‐PKA signalling pathway has been found to rescue hippocampal synaptic defects caused by posttraumatic stress disorder (PTSD).^61^ In this study, we found that Prkaca was markedly downregulated in OGD/R‐induced neurons and MCAO/R rats. Overexpression of Prkaca reduced secretion of inflammatory factors and inhibited OGD/R‐induced Ca^2+^ overload and neuronal apoptosis through activating the cAMP/PKA‐MAPK signalling pathway. And the results of in vivo experiments indicated that overexpression of Prkaca significantly alleviated brain damage in MCAO/R rats.

In summary, this study indicated that DEGs Olfr78 may have a vital role in neuronal apoptosis during CIRI. Mechanism study found that Olfr78 inhibited Ca^2+^ overload and neuronal apoptosis in MCAO/R rats by promoting Prkaca‐mediated activation of cAMP/PKA‐MAPK signalling pathway, therefore, ameliorated brain injury. Our findings may provide new insights into the complex pathophysiological mechanisms of stroke. This study has some limitations. All data we analysed were from online databases and rodents. Further studies need more clinical data to confirm our results. In addition, the potential regulation mechanism of Olfr78 and Prkaca in ischemic brain injury also requires more in‐depth exploration.

## AUTHOR CONTRIBUTIONS

**Tao Kang:** Conceptualization (equal); writing – original draft (equal); writing – review and editing (equal). **Lijuan Zhu:** Conceptualization (equal); writing – review and editing (equal). **Yanli Xue:** Conceptualization (equal); data curation (equal); writing – review and editing (equal). **Qian Yang:** Conceptualization (equal); data curation (equal); writing – review and editing (equal). **Qi Lei:** Conceptualization (equal); writing – review and editing (equal). **Qianqian Wang:** Conceptualization (equal); writing – review and editing (equal).

## FUNDING INFORMATION

This study was supported by the Funding project of basic research of natural sciences in Shaanxi Province (Project No.: 2022JM‐590).

## CONFLICT OF INTEREST STATEMENT

The authors declare that there are no competing interests.

## CONSENT FOR PUBLICATION

All authors have provided consent for the publication of this manuscript.

## Supporting information


**Figure S1.** Enrichment analysis of MCAO/R‐related DEGs in GSE97537 database.
**Figure S2.** Enrichment analysis of MCAO/R‐related DEGs in GSE160500 dataset.
**Figure S3.** Enrichment analysis of overlapping upregulated DEGs in GSE97537 and GSE160500 datasets.
**Figure S4.** Overexpression of Olfr78 inhibits NLRP3 inflammasome activation in OGD/R‐stimulated neurons.
**Figure S5.** Overexpression of Olfr78 or Prkaca alleviates brain injury in MCAO/R rats.
**Figure S6.** Overexpression of Olfr78 or Prkaca inhibits NLRP3 inflammasome activation in MCAO/R rats.
**Figure S7.** Inhibition of Prkaca counteracts the neuroprotective effect of Olfr78 overexpressing in MCAO/R rats.

## Data Availability

The data and materials used and analyzed during the current study are available from the corresponding author on reasonable request.
