# Supplementary material for: Overexpression of olfactory receptor 78 ameliorates brain injury in cerebral ischaemia–reperfusion rats by activating Prkaca‐mediated cAMP/PKA‐MAPK pathway
Source: J Cell Mol Med. 2024 Jun 10;28(11):e18366. doi: 10.1111/jcmm.18366 (PMC11163950; doi:10.1111/jcmm.18366)
Supplement: Supplementary file 1 — Figure S1. Enrichment analysis of MCAO/R‐related DEGs in GSE97537 database. Figure S2. Enrichment analysis of MCAO/R‐related DEGs in GSE160500 dataset. Figure S3. Enrichment analysis of overlapping upregulated DEGs in GSE97537 and GSE160500 datasets. Figure S4. Overexpression of Olfr78 inhibits NLRP3 inflammasome activation in OGD/R‐stimulated neurons. Figure S5. Overexpression of Olfr78 or Prkaca alleviates brain injury in MCAO/R rats. Figure S6. Overexpression of Olfr78 or Prkaca inhibits NLRP3 inflammasome activation in MCAO/R rats. Figure S7. Inhibition of Prkaca counteracts the neuroprotective effect of Olfr78 overexpressing in MCAO/R rats. [file JCMM-28-e18366-s001.docx]

**Supplemental Figure Legends**

**
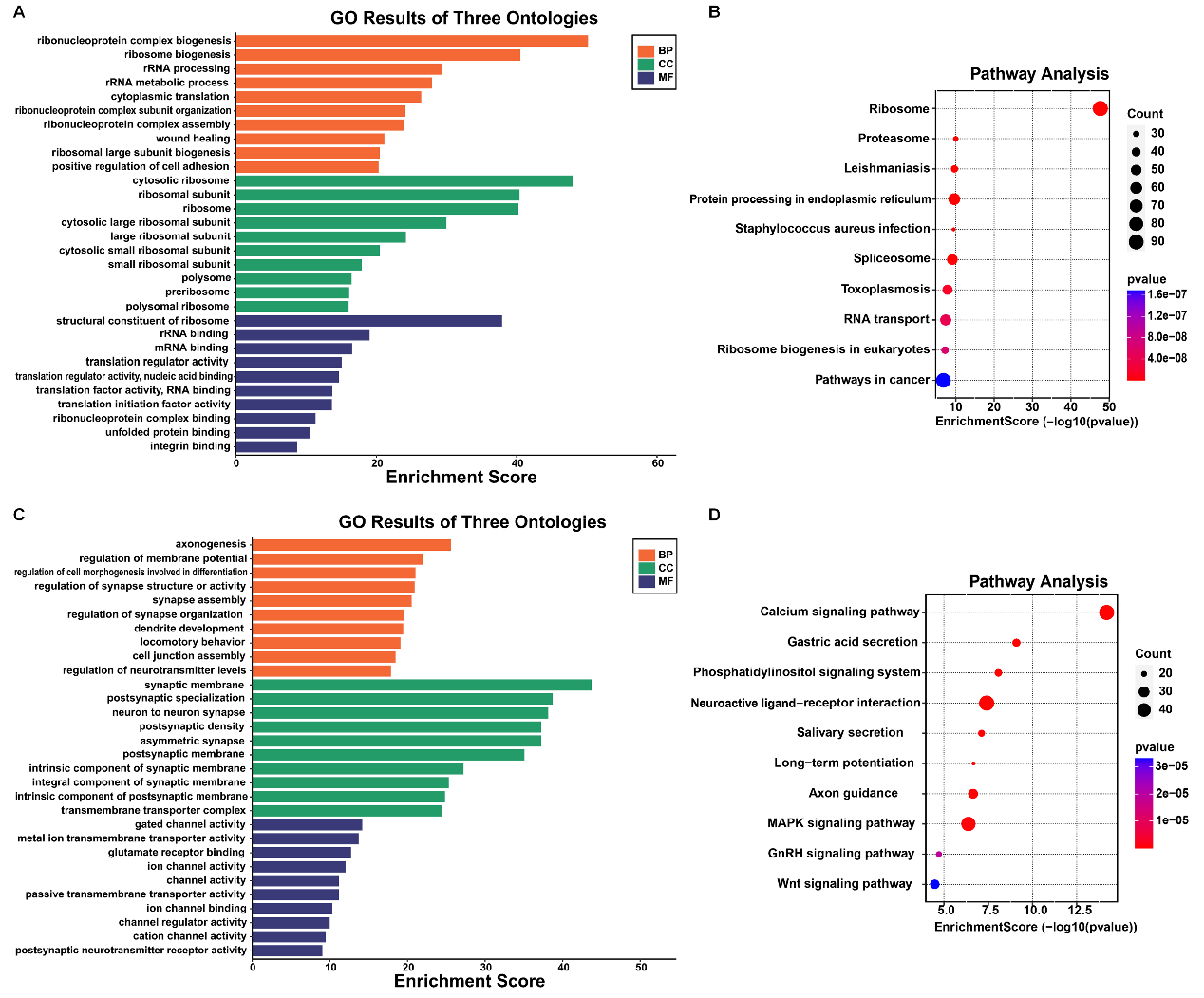
**

**Supplemental Figure 1. Enrichment analysis of MCAO/R-related DEGs in GSE97537 database. A.** GO analysis revealed major enrichment of upregulated MCAO/R-related DEGs in biological processes (BPs), cellular components (CCs), and molecular functions (MFs); **B.** KEGG analysis revealed main enrichment of upregulated MCAO/R-related DEGs in the pathways (*P* value decreases as the color tends to red. The larger the circle, the more genes are enriched); **C.** GO analysis revealed major enrichment of downregulated MCAO/R-related DEGs in biological processes (BPs), cellular components (CCs), and molecular functions (MFs); **D.** KEGG analysis revealed main enrichment of downregulated MCAO/R-related DEGs in the signaling pathways (*P* value decreases as the color tends to red. The larger the circle, the more genes are enriched).

**
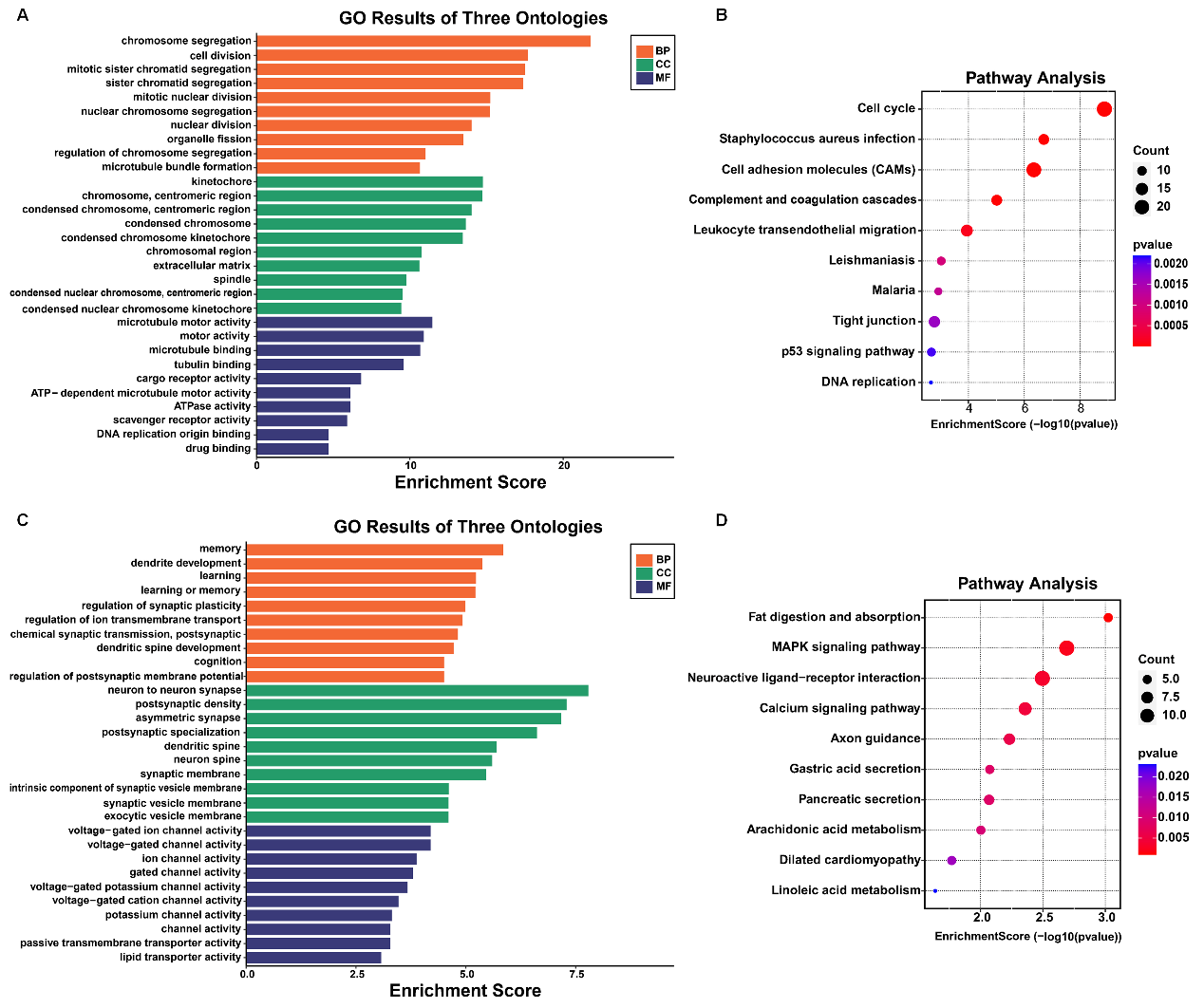
**

**Supplemental Figure 2. Enrichment analysis of MCAO/R-related DEGs in GSE160500 dataset. A.** GO analysis revealed major enrichment of upregulated MCAO/R-related DEGs in biological processes (BPs), cellular components (CCs), and molecular functions (MFs); **B.** KEGG analysis revealed main enrichment of upregulated MCAO/R-related DEGs in the signaling pathways (*P* value decreases as the color tends to red. The larger the circle, the more genes are enriched); **C.** GO analysis revealed major enrichment of downregulated MCAO/R-related DEGs in biological processes (BPs), cellular components (CCs), and molecular functions (MFs); **D.** KEGG analysis revealed main enrichment of downregulated MCAO/R-related DEGs in the signaling pathways (*P* value decreases as the color tends to red. The larger the circle, the more genes are enriched).

**
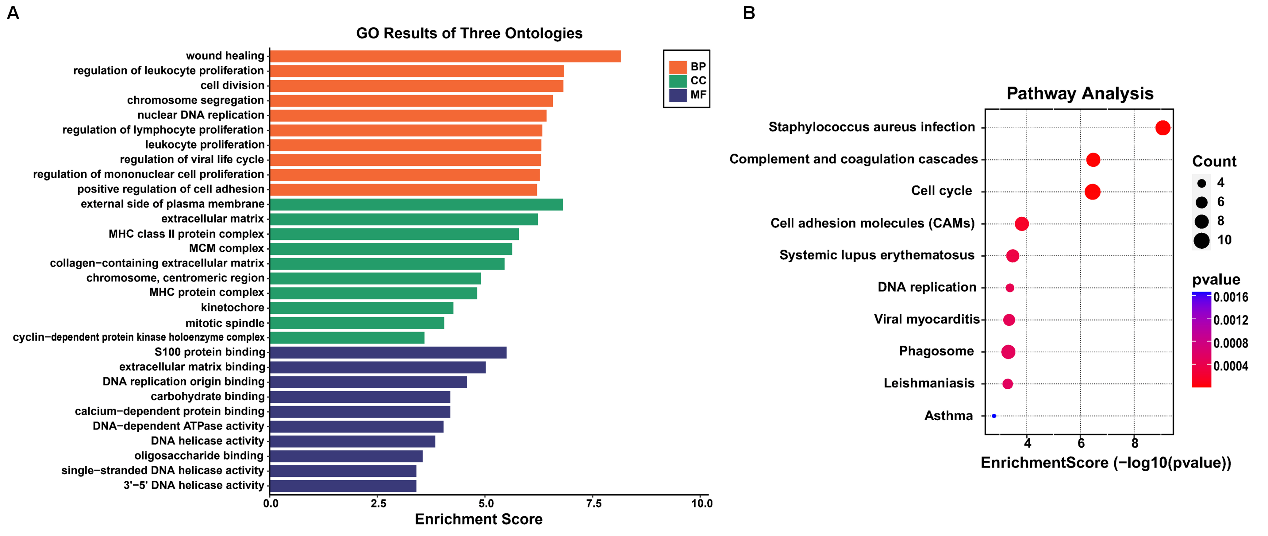
**

**Supplemental Figure 3. Enrichment analysis of overlapping upregulated** **DEGs in GSE97537 and GSE160500 datasets. A.** GO analysis revealed major enrichment of upregulated MCAO/R-related DEGs in biological processes (BPs), cellular components (CCs), and molecular functions (MFs); **B.** KEGG analysis revealed main enrichment of upregulated MCAO/R-related DEGs in the signaling pathways (*P* value decreases as the color tends to red. The larger the circle, the more genes are enriched).

**
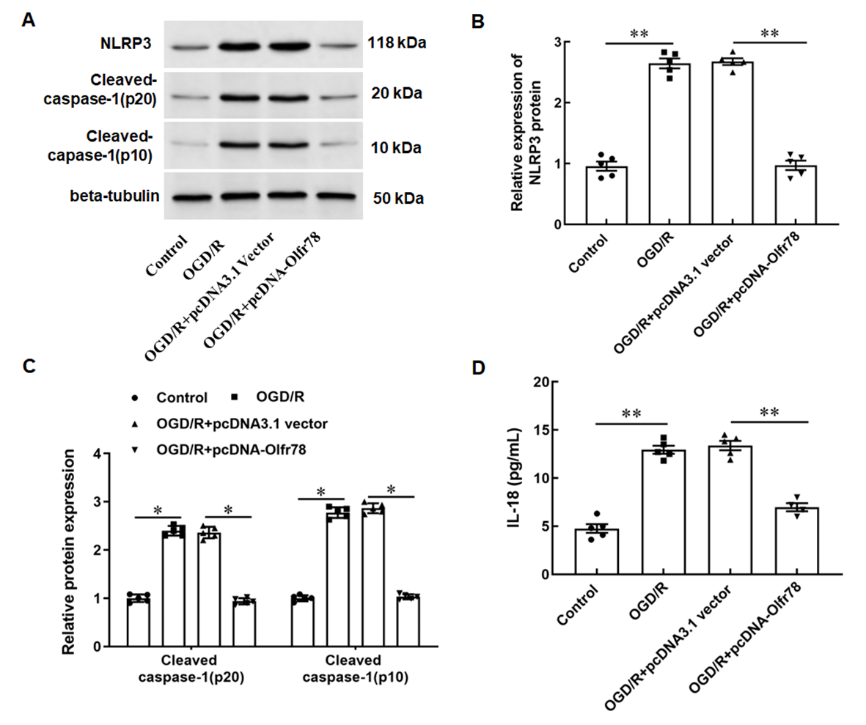
**

**Supplemental Figure 4. Overexpression of Olfr78 inhibits NLRP3 inflammasome activation in OGD/R-stimulated neurons.** Neurons were transfected with Olfr78 overexpression vector (pcDNA-Olfr78) or empty vector (pcDNA3.1 vector), and then treated with OGD/R for 12 h. **A-C.** The protein levels of NLRP3 (B) and Cleaved-caspase-1 (C) were detected with Western blotting. **D.** The secretion of inflammatory factor IL-18 was detected with ELISA. Data are expressed as means ± SEM, n=5. Statistical differences were evaluated by using one-way and two-way ANOV, and followed by Tukey HSD test. Compare with the control group or the OGD/R+pcDNA3.1 vector group, **P* < 0.05, ***P* < 0.01.


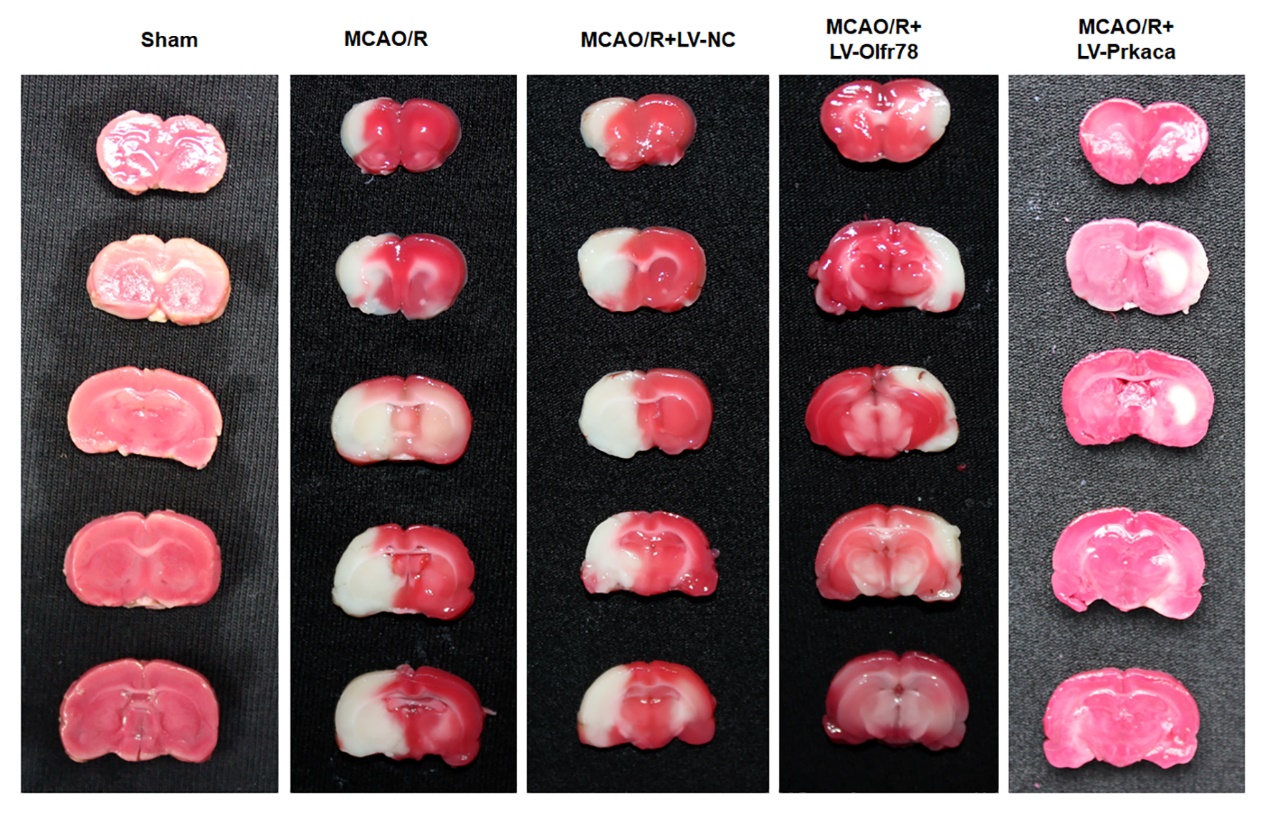


**Supplemental Figure 5. Overexpression of Olfr78 or Prkaca alleviates brain injury in MCAO/R rats.** Forty SD rats were randomly divided into 5 groups: the sham group, MCAO/R model group, MCAO/R+LV-NC group, MCAO/R+LV-Olfr78 group, and MCAO/R+LV-Prkaca group, with 8 rats in each group. TTC staining of brain tissues of rats in each group.


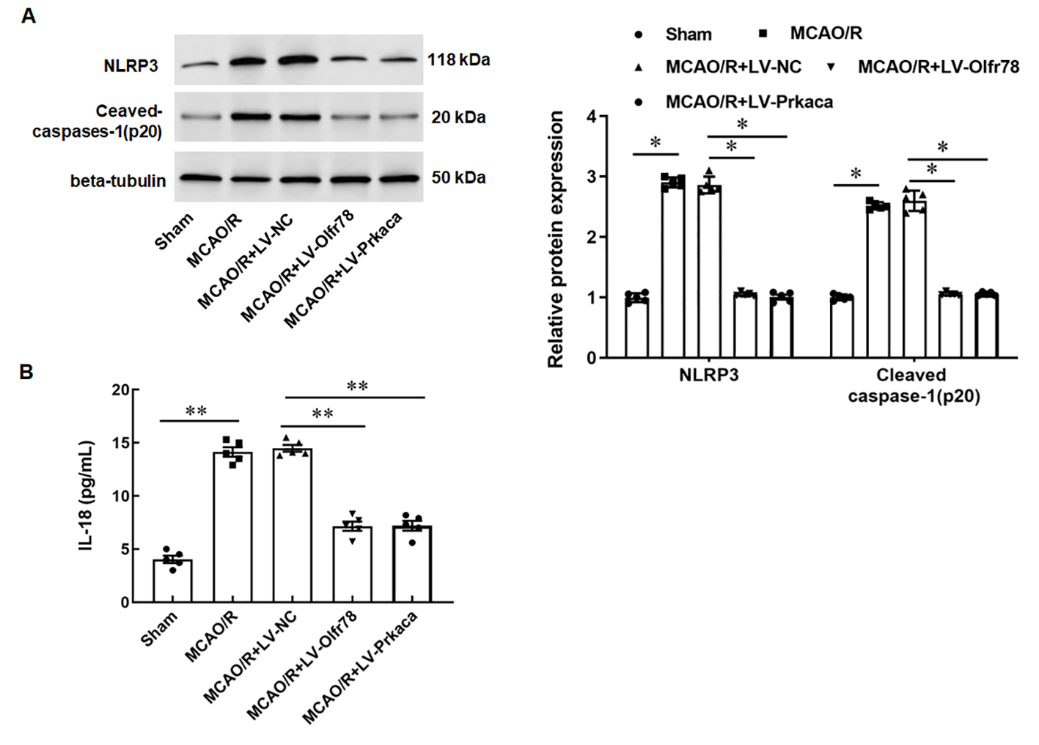


**Supplemental Figure 6. Overexpression of Olfr78 or Prkaca inhibits NLRP3 inflammasome activation in MCAO/R rats.** Forty SD rats were randomly divided into 5 groups: the sham group, MCAO/R model group, MCAO/R+LV-NC group, MCAO/R+LV-Olfr78 group, and MCAO/R+LV-Prkaca group, with 8 rats in each group. **A.** The protein levels of NLRP3 and Cleaved-caspase-1 were detected with Western blotting. **B.** The secretion of inflammatory factor IL-18 was detected with ELISA. Data are expressed as means ± SEM, n=5. Statistical differences were evaluated by using one-way, and followed by Tukey HSD test. Compare with the sham group, MCAO/R group, or the MCAO/R+LV-NC group, **P* < 0.05, ***P* < 0.01.


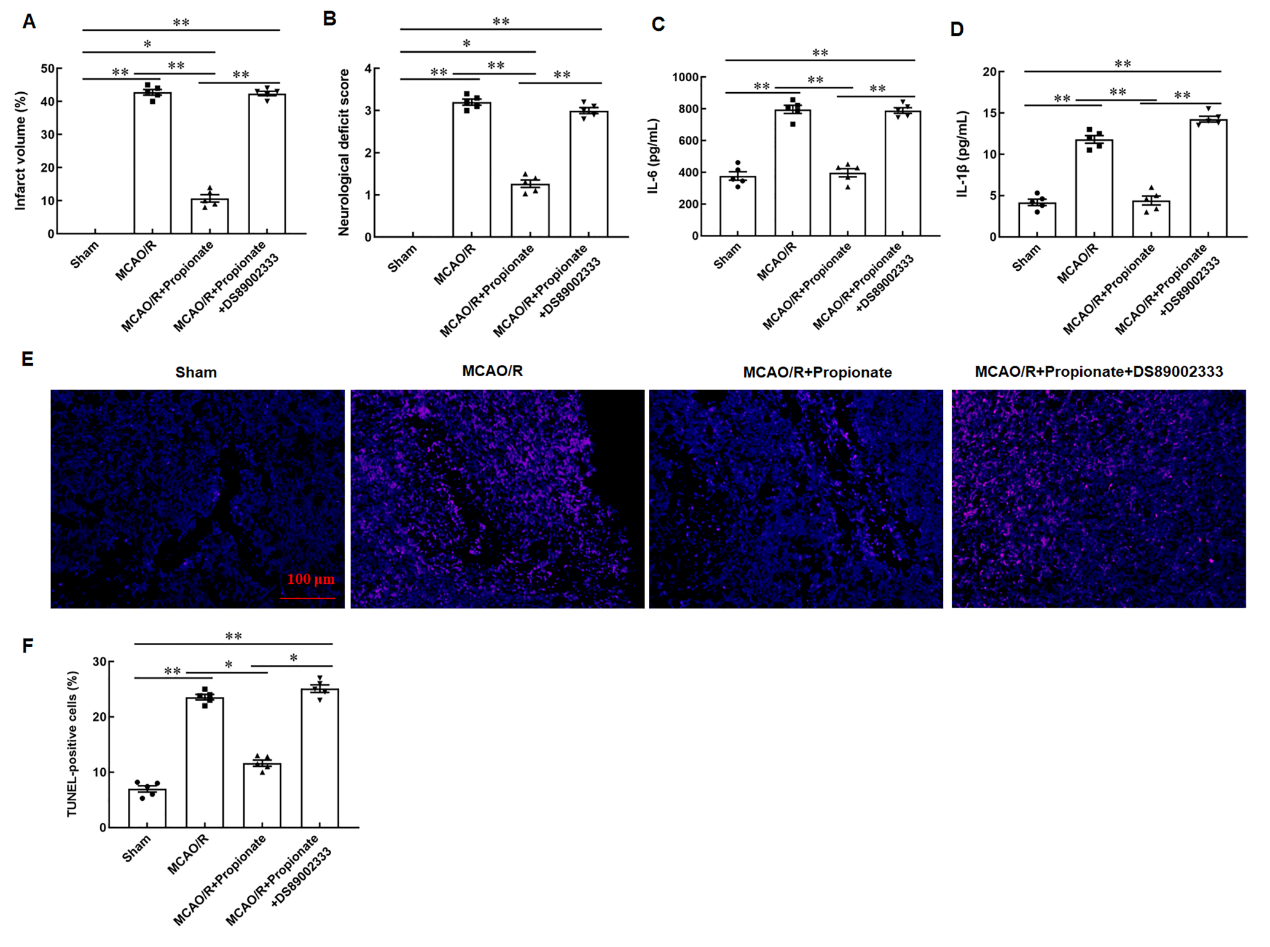


**Supplemental Figure 7. Inhibition of Prkaca counteracts the neuroprotective effect of Olfr78 overexpressing in MCAO/R rats.** Thirty-two SD rats were randomly divided into 4 groups: the sham group, MCAO/R model group, MCAO/R+ Propionate group, and the MCAO/R+ Propionate+ DS89002333 group, with 8 rats in each group. **A.** Statistics of infarct volume of brain tissue of rats in each group; **B.** Statistics of neurological deficit scores of rats in each group. **C-D.** The concentration of IL-6 (C) and IL-1β (D) in peripheral blood of rats in each group was detected with ELISA; **E-F.** Neuronal apoptosis in brain tissues of rats in each group was detected with TUNEL staining. Data are expressed as means ± SEM, n=5. Statistical differences were evaluated by using one-way, and followed by Tukey HSD test. Compare with the sham group, MCAO/R group, or the MCAO/R+ Propionate group, **P* < 0.05, ***P* < 0.01.
